# The cost of the diagnostic odyssey of patients with suspected rare diseases

**DOI:** 10.1186/s13023-025-03751-y

**Published:** 2025-05-10

**Authors:** Rick Glaubitz, Luise Heinrich, Falko Tesch, Martin Seifert, Katrin Christiane Reber, Ursula Marschall, Jochen Schmitt, Gabriele Müller

**Affiliations:** 1 Center for Evidence-Based Healthcare, Faculty of Medicine and University Hospital Carl Gustav Carus, Technische Universität Dresden, Dresden, Germany; 2 AOK Nordost. Die Gesundheitskasse, Health Services Management, Berlin, Germany; 3 Department of Medicine and Health Services Research, BARMER Health Insurance, Wuppertal, Germany

**Keywords:** Rare diseases, Diagnostic pathway, Diagnostic costs, Utilization

## Abstract

**Purpose:**

Patients with rare diseases often undergo a long diagnostic odyssey. However, there is little empirical evidence on the cost incurred during the diagnostic pathway for patients with suspected rare diseases. This study provides a comprehensive analysis of healthcare costs and utilization during the diagnostic pathway for a heterogeneous sample of patients with suspected rare diseases but unclear diagnosis.

**Methods:**

Using claims data from five German statutory health insurance organizations for the years 2014–2019, we analyzed costs and healthcare utilization of 1,243 patients (aged 0 to 82 years) with suspected rare diseases referred to a rare disease center. A control cohort was assigned using 1:75 exact matching on age, sex and place of residence.

**Results:**

In the years prior to referral to an expert center, healthcare utilization of patients with suspected rare diseases was, on average, substantially and significantly higher compared to a matched control cohort during the same observation period – e.g. in terms of the number of hospitalizations (3.1 (95%CI: 2.9–3.4) vs. 0.5 (95%CI: 0.5–0.5)), different diagnoses (50.0 (95%CI: 48.1–51.9) vs. 26.4 (95%CI: 26.2–26.5)), different active substances prescribed (12.7 (95%CI: 12.2–13.3) vs. 8.2 (95%CI: 8.2–8.3)) and the number of genetic tests (14.7 (95%CI: 12.6–16.7) vs. 0.3 (95%CI: 0.3–0.3)). We found evidence of heterogeneity in utilization by age and sex. On average, direct costs (inpatient, outpatient and prescription drug costs) of patients with suspected rare diseases during the diagnostic pathway were 7.6-fold higher than the costs of matched controls (€26,999 (95%CI: €23,751 − 30,247) vs. €3,561 (95% CI: € 3,455-3,667)). Inpatient costs were the main cost component, accounting for 62.5% of total costs.

**Conclusions:**

The diagnostic odyssey of patients with suspected rare diseases is associated with extensive healthcare utilization and high cost. Against this background, new ways to shorten the diagnostic journey have a high potential to decrease the financial burden related to rare diseases.

**Supplementary Information:**

The online version contains supplementary material available at 10.1186/s13023-025-03751-y.

## Introduction

Rare diseases are a major challenge for health care systems around the world. To date, it is estimated that over 300 million people worldwide are affected by rare diseases, of which more than 6,000 rare diseases have already been identified, with approximately 72% of these diseases being of genetic origin [1].

In addition to the severe health conditions reflected in increased morbidity and premature death [2, 3], those affected by rare diseases diseases are difficult to diagnose, leading to many patients undergoing a lengthy diagnostic journey accompanied by frequent changes in physicians, misdiagnosis, and inappropriate treatment – a process termed the “diagnostic odyssey” [4–7]. Diagnostic delay and misdiagnoses along the way are associated with adverse disease-related health outcomes [8, 9] and mental health burdens both for those directly affected by an undiagnosed disease and their families [9–11].

Moreover, rare diseases come with a high economic burden for affected patients, their families and the healthcare systems as a whole. There is a limited but growing body of cost-of-illness studies assessing the, on average, high economic costs associated with rare disease patients for different rare diseases and countries [12, 13]. However, generally, those studies focus on patients already diagnosed with a rare disease. Comprehensive studies on the costs incurred during the diagnostic odyssey are scarce. The few existing studies clearly point towards high diagnostic costs [6, 14–18]. However, their external validity is limited by their small population size or the exclusive focus on certain age groups (often infants or children) and specific rare diseases. Neglecting diagnostic costs and therefore the costs of undiagnosed rare disease patients leads to an underestimation of the actual economic burden of rare diseases. Against this background, recent related studies have highlighted the importance of studying the costs incurred during the diagnostic journey of patients suffering from rare diseases [13, 19, 20].

In this study, we address this need for comprehensive analyses of the diagnostic pathway and its related costs by examining a rich set of diagnostic and therapeutic services and their costs for patients with suspected but undiagnosed rare diseases based on statutory health insurance (SHI) claims data for Germany. Their utilized services and costs are then compared to an age-, sex- and place of residence-matched control cohort for the same observation period to estimate the direct costs of the diagnostic odyssey and identify main cost drivers.

## Material and methodology

### The TRANSLATE-NAMSE project

Our health economic evaluation of the diagnostic journey of individuals with suspected rare diseases is part of the Innovation Fund project *TRANSLATE-NAMSE* (TNAMSE), which was funded by the German Federal Joint Committee (G-BA; grant number: 01NVF16024) from 2017 to 2020. Aim of this project was the establishment of better care structures by supporting the implementation of key measures of the German National Plan of Action for People with Rare Diseases, which was established in 2013 on initiative of the National Action League for People with Rare Diseases (NAMSE).

One of the main care deficits that the project aimed to address was the delayed diagnosis of rare disease patients. To shorten the time to diagnosis and improve subsequent treatment of these patients, the establishment of centers for rare diseases at university hospitals was one of the main demands of the NAMSE Action Plan. In this context, the TNAMSE project assessed the performance of nine German centers for rare diseases (Berlin, Bonn, Dresden, Essen, Hamburg, Heidelberg, Lübeck, Munich, Tübingen) in ending patients diagnostic odyssey with the help of interdisciplinary case conferences and exome diagnostic tests [21].

Specifically, our study is based on the part of the TNAMSE project that aimed to enhance comprehension of the diagnostic process and its associated costs for patients seeking a diagnosis for a suspected rare disease. Further information on the TNAMSE project can be found in the project report [22] or existing studies focusing on other parts of the TNAMSE project [21, 23, 24].

### Data

We used health insurance data provided by five German SHI providers (BARMER, AOK-Nordost, AOK Bayern, AOK PLUS and AOK Baden-Württemberg). For four providers, data were available for the years 2014 to 2019 while one health insurance provided us with data for the years 2015 to 2019. The data contained detailed information on:

- inpatient and outpatient diagnoses (ICD-10-GM codes; International Statistical Classification of Diseases—German Modification),
- medical procedures and treatments according to OPS classification (German adoption of ICMP) and the German outpatient procedure classification system EBM (“Einheitlicher Bewertungsmaßstab”) and their associated costs,
- medical prescriptions (ATC codes) and their associated costs,
- the length of hospital stays (in days) and.
- sociodemographic information (age, sex, date of death, insurance periods, place of residence).

Information on the first occurrence of TNAMSE-related symptoms was obtained from a project-specific patient questionnaire. We were able to link this information to patients’ health insurance data via a project-specific patient ID.

### Study population

In our study, we analyzed patients with an unclear diagnosis who contacted one of the nine German centers for rare diseases participating in the TNAMSE project. Unclear diagnosis refers to patients showing unclear clinical pictures pointing towards a high probability of suffering from a rare disease, but their present symptoms do not allow to derive a clear diagnosis or main criteria of the diagnosis are not fulfilled or additional significant symptoms that are typical for the diagnosis are not existing [25]. In addition, the full range of conventional investigations to rule out existing suspected diagnoses should have been carried out prior to enrolment in the project. However, it is important to note that due to the design of the TNAMSE project, we did not only include patients who were ultimately diagnosed with a rare disease, but also those with a common disease or for whom no diagnosis was made within the TNAMSE project. In this analysis, we excluded those individuals participating in the TNAMSE project that met at least one of the following two exclusion criteria:

1. No health insurance data available (not a member of one of the five participating health insurance providers or insurance data not identifiable by insurance number).
2. Less than 35 control patients identified.

The application of the first exclusion condition reduced the number of TNAMSE patients available for economic analysis by around 73% (*N* = 3,515), while the second exclusion condition reduced the sample by less than 1% (*N* = 43). 1 There were no relevant differences between TNAMSE patients available for economic analysis and excluded TNAMSE patients.^2^ After applying these restrictions, 1,243 patients (out of originally 4,801) aged 0 to 82 in the years 2014 to 2019 were available for our analysis. Overall, 313 patients (25.2%) ended up diagnosed with a rare disease, 33 (2.7%) with a common disease, 16 (1.3%) with a psychosomatic disorder and for 881 (70.9%) no diagnosis was identified within the TNAMSE project.^3^ In our baseline analysis, we pooled all these patients in order to reach a sample size large enough to allow for stratification by age (categories) and sex.^4^

### Matching procedure

Since it is the aim of this study is to examine additional diagnostic procedures and consequently additional costs from the SHI perspective for patients with undiagnosed diseases, we need some sort of control group mirroring the average costs of an equivalent patient without an undiagnosed disease. Using a matched cohort approach, we aim to estimate sex-, region- and age-specific expected costs and diagnostics that would have incurred in the absence of an undiagnosed condition. A matched control cohort was assigned from health insurance data using exact matching on age, sex and place of residence (first three digits of 5-digit-ZIP code).^5^ Place of residence was used as a matching variable since there is evidence for regional utilization of health services which also depends on regional supply [26, 27]. In addition, information on matched controls had to be available for the entire observation period of their matched TNAMSE patient. We aimed to match 75 controls to each TNAMSE patient. This number of assigned controls is considerably larger than in most existing claims-data-based matched cohort studies [28]. However, we followed existing studies using a larger number of matched controls in order to mitigate the risk of under- or overestimation of average costs of (control) patients without undiagnosed diseases [29, 30]. This is of particular importance for our analysis as patients with undiagnosed rare diseases were not clearly identifiable in the health insurance claims data and therefore could not be excluded from the pool of potential controls.^6^ We applied matching with replacement.

In total, 92,078 controls were matched to the 1,243 included TNAMSE patients – an average of 74.1 controls per person with an undiagnosed disease (Minimum: 35; Maximum: 75).^7^ As shown in Table 1, there were no significant differences in sex and age between the two groups after matching.

**Table 1:** Characteristics of TNAMSE patients and matched control cohort

Indicator	Category	TNAMSE patients		Matched cohort	
		*N*	(%)	*N*	(%)
*N*		1,243		92,078	
**Sex**	Men	671	54%	49,697	54%
	Women	572	46%	42,381	46%
**Age groups**	< 1 year	138	11%	9,492	10%
	1–17 years	822	66%	61,416	67%
	≥ 18 years	283	23%	21,170	23%
		**Mean ± SD**	**Median**	**Mean ± SD**	**Median**
**Age (in years)**		14.8 ± 18.4	7	15.0 ± 18.4	8

### Statistical analysis and cost calculations

After matching, we calculate the average additional costs by subtracting the average costs of the control group from the costs of the TNAMSE patients. In a similar way, we calculated differences in the healthcare utilization (e.g. the number of hospitalizations, different diagnoses and the number of genetic tests) for TNAMSE patients and their matched control group.

Treatment costs (inpatient and outpatient) and costs of medical prescriptions were analyzed from the SHI perspective. Out-of-pocket payments and co-payments were not taken into account. In our baseline analyses, we examined the (average) total costs per patient for their entire observation period.^8^ The patient-specific observation period started with the first occurrence of symptoms (for which the patient later contacted the center for rare diseases) and ended with the patient’s TNAMSE start date. However, due to limitations in data availability, our observation period was limited to the years between 2014 (2015 for one SHI provider) and 2019. Information on the first occurrence of TNAMSE-related symptoms was obtained from a project-specific patient questionnaire. If information on the duration of symptoms was missing (54.5% of our included 1,243 patients), all available data for the years 2014 to 2019 were used. The same applied if the first symptoms already occurred before health insurance data were available (i.e. before 2014 or 2015) – which was the case for 18.7% of our 1,243 included TNAMSE patients.

For the descriptive analyses of costs and diagnostics, we derived means (with standard deviations), 95% confidence intervals (95% CIs) and medians. Non-overlapping 95% CIs depict significant differences between comparable groups [31, 32]. In our baseline analyses, we stratified individuals by sex and age at inclusion in the TNAMSE project (younger than 1 year; 1–17 years old; 18 years or older). All statistical analyses were conducted using Stata (version 15.1).

## Results

### Diagnostic pathways

In general, the utilization of healthcare services by patients with suspected but undiagnosed rare diseases differs substantially from that of people without such diseases.

**Fig. 1:**
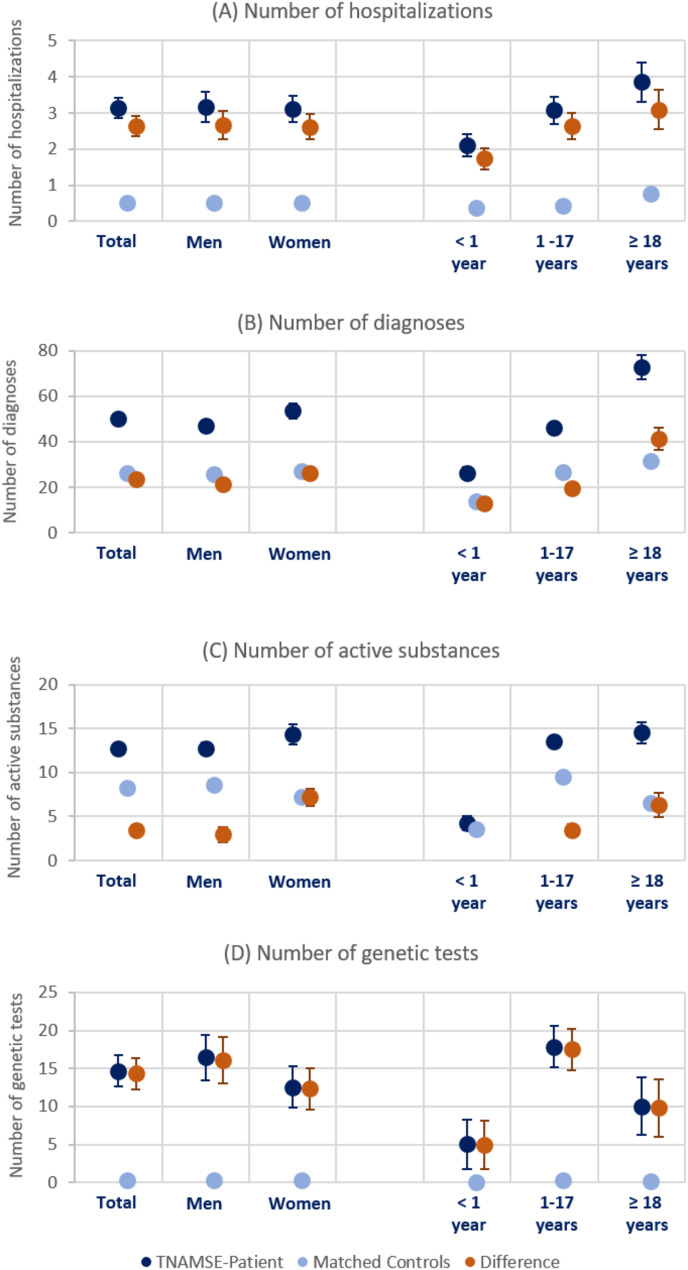
Diagnostic pathway indicators, by sex and age. Note: Means with 95% confidence intervals for TNAMSE patients, matched controls and the difference between these two groups

Figure 1 (Panel A) depicts the average number of hospitalizations of both the TNAMSE patients (dark blue) and their matched controls (light blue), as well as the mean difference between these two groups (orange). During the same observation period, on average, TNAMSE patients were hospitalized on average 3.1 (95%CI: 2.9–3.4) times, while individuals without a suspected rare disease were hospitalized 0.5 (95%CI: 0.5–0.5) times. While there were no clear differences by sex, the average number of hospitalizations increased with age.^9^ Furthermore, as shown in Table A2 in the Appendix, the average duration of hospital stays was significantly higher for TNAMSE patient than for their matched controls (Mean difference: 19.2 days; 95%CI: 16.6–21.8).^10^ In particular, infants (age < 1 year) with a suspected rare disease spent significantly more time in hospital than their matched controls.

A more frequent change of diagnosis is to be expected for patients with an unclear disease. Therefore, we analyzed the number of different 4-digit ICD-10 codes documented for both inpatient and outpatient care during the observation period. For the matched control cohort, an average of 26.4 (95%CI: 26.2–26.5) diagnoses per insured person were found (Fig. 1, Panel B). For TNAMSE patients, this value was almost twice as high at 50.0 (95%CI: 48.1–51.9) diagnoses. We found evidence that the number of additional diagnoses for patients with an undiagnosed disease increased with age. The search for a diagnosis was also reflected in the number of outpatient specialists consulted (Table A4, Appendix A).^11^ While the matched control cohort consulted an average of 4.3 (95%CI: 4.3–4.4) specialists, the TNAMSE patients consulted an average of 7.3 (95%CI: 7.0-7.5) specialists during the same observation period. There were differences not only by age but also by sex with female patients consulting significantly more specialists than male patients. We found similar patterns when analyzing the number of different outpatient facilities as an indicator for the search for a diagnosis (Table A4, Appendix A).^12^

Next, we analyzed the number of different active substances prescribed using claims data on 5-digit ATC codes. Specifically, our data source included information on all outpatient prescriptions filled at a pharmacy. The higher number of different active substances prescribed to TNAMSE patients reflected not only the increased need for treatment, but also the attempt to find suitable treatment with different drugs. On average, 8.2 (95%CI: 8.2–8.3) different active substances were prescribed to the cohort of control patients during the observation period (Fig. 1, Panel C). In contrast, TNAMSE patients received an average of 12.7 (95%CI: 12.2–13.3), a significantly higher number of active substances. Due to long hospital stays, infants (< 1 year of age) received comparatively fewer different active substances directly from pharmacies.

Furthermore, we investigated the use of different diagnostic procedures (imaging procedures, biopsies, genetic testing and laboratory diagnostics), as these are often particularly cost-intensive from the perspective of SHI providers. As the majority of rare diseases are genetic, exome sequencing is an important diagnostic method for patients with an unclear diagnosis. However, at the time of the TNAMSE project, these tests were reimbursed by health insurance providers only in rare cases and after a separate request for reimbursement. During the study period, requests for genetic tumor diagnostics in particular were eligible for reimbursement. However, single gene and gene panel sequencing was already covered by SHI providers and was therefore available for investigation. Specifically, Panel D of Fig. 1 depicts the (average) sum of all fee schedule items (GOP) of the German Uniform Assessment Standard (EBM) that begin with the two digits “11” and refer to different genetic tests.^13^ On average, 14.7 (95%CI: 12.6–16.7) human genetic GOPs were billed for individuals with suspected rare diseases before their inclusion in the TNAMSE project. Among the matched controls, there were only 0.3 (95%CI: 0.3–0.3) GOPs during the same observation period, and thus significantly fewer genetic tests. Genetic testing was most common in patients aged between 1 and 17 years. Infants had a comparatively lower number of GOPs, as these tests, if required, were mainly performed in a clinical context or as part of research projects.

Additionally, Tables A6, A7 and A9 in Appendix (A) report results for other high-cost diagnostic procedures such as biopsies, imaging procedures and laboratory tests.^14^ For all these procedures, we found evidence for, on average, more frequent applications among TNAMSE patients compared to their matched cohort. For all these diagnostic procedures, use was particularly high among older patients (aged 18 years and older).

### Cost analysis

In the next step, we analyzed the costs of the extensive diagnostic pathway of patients with undiagnosed but suspected rare diseases.^15^

**Fig. 2:**
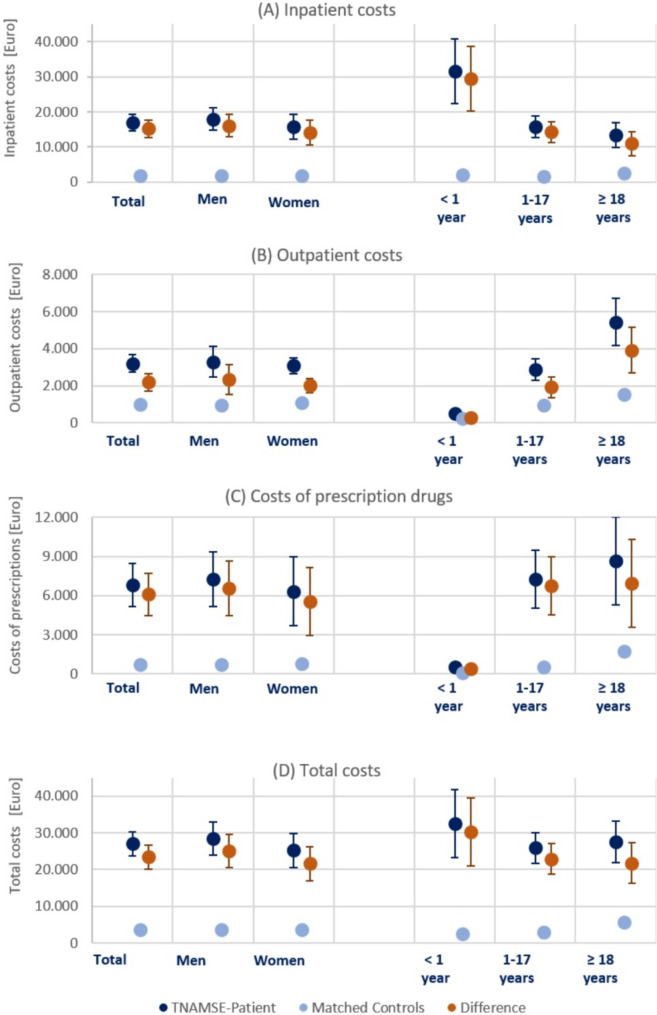
Diagnostic cost components, by sex and age. Note: Means with 95% confidence intervals for TNAMSE patients, matched controls and the difference between these two groups. Costs in Euro

Figure 2 (Panel A) depicts the inpatient costs (in Euro) of both TNAMSE patients and their matched controls. The average inpatient costs for TNAMSE patients amounted to €16,983 (95%CI: €14,585 − 19,380), which was about 9.4-fold higher than the inpatient costs that would be expected for individuals without an undiagnosed (rare) disease during the same observation period (Mean: €1,809; 95%CI: €1,735-1,883). The highest costs occurred for infants (< 1 year) and average inpatient costs decreased with age.

As shown in Fig. 2 (Panel B), the outpatient costs of TNAMSE patients were also significantly higher than the outpatient costs of their matched control cohort. On average, the outpatient costs of a TNAMSE patient exceeded the expected costs by € 2,194 (95%CI: € 1,726-2,661). The low costs for infants (< 1 year) were due to the limited period of observation (Mean: 310 ± 130 days), which by design was less than one year. With increasing age, outpatient costs increased sharply, which was partly due to the longer (average) observation period.^16^

The costs of outpatient prescriptions filled at a pharmacy over the entire observation period for all TNAMSE patients, as depicted by Fig. 2 (Panel C), were on average €6,820 (95%CI: €5,171-8,468). In contrast, the costs for the matched cohort were significantly lower (Mean: €741; 95%CI: €675–808). Thus, on average, the drug costs of a TNAMSE patient exceeded the expected costs by 9.2-fold, or €6,085 (95% CI: €4,438-7,732). The very low costs for infants were due to their short observation period and the comparatively long inpatient stays (see Fig. 1), where the medication costs were included in the inpatient costs. Interestingly, the difference in the median costs of outpatient prescriptions was much smaller and even negative (-€44) for individuals aged 18 and older. This finding might be an indication that (drug) therapy of individuals with undiagnosed diseases is often insufficient.

Finally, Panel D of Fig. 2 presents the total costs which are the sum of inpatient costs, outpatient costs, and the costs of prescriptions filled at pharmacies. Total costs of TNAMSE patients over the entire observation period amounted to an average of €26,999 (95%CI: €23,751 − 30,247). For the matched cohort, total costs were on average €3,561 (95% CI: € 3,455-3,667). Thus, the total costs of a TNAMSE patient exceeded the expected costs by an average of €23,453 (95%CI: € 20,221 − 26,685). On average, total costs were particularly high for very young TNAMSE patients (< 1 year).

## Discussion

Using German claims data, our study comprehensively confirms and quantifies the long and extensive diagnostic pathway of patients with (undiagnosed) rare diseases found in related studies [4–7]. Specifically, we found evidence of an extensive diagnostic odyssey characterized by, for example, a significantly higher number of hospitalizations, different diagnoses, active substances prescribed and diagnostic tests compared to a matched cohort. However, compared to existing studies our relatively large and heterogeneous study population provides new insights into differences by age and sex.

We found significant differences in utilization by age and sex. For example, we found evidence that the average duration of inpatient stays was significantly longer for infants than for older patients, while the average number of genetic tests was highest for patients aged between 1 and 17 years. On average, healthcare utilization during the diagnostic journey was significantly higher for women than for men for a number of indicators (e.g. number of diagnoses, number of consulted specialists), while for others there are no clear sex differences (e.g. number of hospitalizations). Differences by sex regarding the diagnostic pathway could potentially explain the recent evidence for longer average diagnostic journeys for women [7].

Moreover, our study offers a comprehensive picture not only on the extent of the diagnostic journey of patients with suspected but undiagnosed rare diseases but also the associated inpatient and outpatient costs. In our sample, mean direct costs were about 7.6-fold higher (€26,999 vs. €3,561) than what would be expected for individuals without a suspected rare disease during the same observation period. The main cost component across all age groups and for both men and women was inpatient costs, which on average accounted for around 62.5% of total costs. However, the level of inpatient costs varied between age groups. Despite the substantially shorter average observation period, infants’ average inpatient costs were significantly higher than for patients aged 1–17 years and the group of patients aged 18 years and older. This was due to the particularly cost-intensive neonatal care and the long average duration of hospital stays (33.0 ± 39.4 days). On average, inpatient costs represented 96.3% of total costs for infants in our sample. For the two older age groups (1–17 years and ≥ 18 years), inpatient costs represented 60.5% and 48.4% of total costs, respectively. In contrast to infants, they were more likely to utilize outpatient services on their search for a diagnosis and the treatment of illness-related medical conditions, resulting in significantly higher outpatient costs and pharmacy prescription costs. As a result, total costs did not vary significantly between the different age groups. Furthermore, we found no evidence of significant differences by sex in total costs and their main drivers.

Direct comparisons of our findings on the cost of the diagnostic odyssey with those of the few other related studies [6, 14–18] are hampered by differences in the populations analyzed (e.g. age groups, specific types of rare diseases), observation periods, cost components, study design and methodology, and country-specific characteristics.^17^ Nevertheless, our results are broadly consistent with other studies in terms of supporting the general notion that costs incurred during the diagnostic pathway are substantial.

By highlighting the significant cost associated with the search for a diagnosis, our study emphasizes the need to shorten the diagnostic odyssey – not only for the sake of the patients, but also for reasons of cost efficiency. In recent years, a number of specific programs dedicated to improving the diagnosis of rare disease have been established [33]. In general, there is evidence that rapid referral to experts (e.g. at rare disease centers) has a positive impact on the diagnostic process and shortens the time to correct diagnosis [7, 34, 35]. Willmen et al. 2021 [18] found a large cost-saving potential of rapid referral to expert centers by showing that the majority of total diagnostic costs (around 75%) occur before a patient’s referral and only about 25% of costs occur during treatment and successful diagnosis at the expert center. In addition, a growing number of studies point to the benefits of diagnostic support systems in reducing delayed diagnosis and diagnostic costs [18, 36]. Furthermore, recent studies supported the early use of genomic testing (e.g. whole-exome sequencing (WES) and whole-genome sequencing (WGS)) to provide faster and more cost-effective diagnoses compared to standard diagnostic approaches [15, 19, 37–40]. However, these studies on the cost-effectiveness of next-generation sequencing (NGS) genomic testing were primarily based on analyses of infants and children, while evidence on adults is lacking.

### Strengths and limitations

The main strength of our study is the rich set of diagnostic indicators and different direct cost components available in our health insurance claims data, which allowed us to describe the diagnostic odyssey in a comprehensive way without recall bias. In addition, our study population is relatively large compared to those of existing studies, leading to more precise estimates and allowing for stratification by age and sex.

Despite providing important new insights, our study suffers from some limitations. First, similar to the challenges encountered in a related study for Australia [41], our analysis was hampered by the lack of a specific International Classification of Disease (ICD) 10 code for many rare diseases, which prevented the clear identification of individuals with rare diseases in SHI claims data.^18^ Therefore, in theory, there was no guarantee that individuals in the matched cohort did not have a rare disease themselves. For this reason, we matched a large number of controls (1:75 matching) to reduce the risk of under- or overestimating the average costs of matched individuals without undiagnosed conditions. While our study only focused on the “average patient” (matched by age, sex and place of residence) as the control group, future studies could expand this analysis. For example, since rare disease are often chronic conditions, one could also compare costs of patients with suspected but undiagnosed rare diseases with those of patients with non-rare chronic conditions.

Second, we had to limit the observation period for the diagnostic odyssey to a maximum of six years (2014–2019), as health insurance providers are restricted by regulatory requirements to not retain personal data for longer periods. However, prior studies have shown that the variation in time to diagnosis is extensive and for many patients the diagnostic process can take more than a decade [4, 45, 46]. TNAMSE patients reported, on average, 6.9 (± 8.5) years between the first occurrence of symptoms and their TNAMSE start date, exceeding our maximum observation period. Furthermore, older individuals (≥ 18 years) reported an even longer average duration of their diagnostic journey (10.4 ± 11.4 years). Against this background, our estimates of the total (direct) costs of the diagnostic odyssey until referral to an expert center should be interpreted as a lower bound estimate. A simple exemplary calculation for individuals aged 18 years or older using their average annual costs of €7,081 (Table A13 in Appendix A) and multiplying it by the age-specific average duration of their diagnostic journey (10.4 years) yields estimated total costs of €73,642, which is substantially higher than our baseline total cost estimate for this age group of €27,513 (95% CI: €21,922 − 33,103). Information on the first occurrence of disease-related symptoms, which marked the start of our observation period where available, was self-reported and may therefore be subject to recall bias.

In addition, a detailed description of our study population was hampered by the fact that it is a heterogeneous group of patients with a wide variety of different health conditions. It remains unclear whether our study population is representative of the general population of patients seeking a diagnosis for a suspected but so far undiagnosed rare disease. 19 Data was only available on the insured for a limited number of different regional SHI providers, which may lead to over- or underrepresentation of patients from certain regions. Besides, patients were not randomly selected to participate in the TNAMSE project, which could be a source of selection bias. For example, as the TNAMSE project only included patients who have contacted one of the participating German centers for rare diseases, our sample was more likely to include patients who were themselves particularly committed to ending the diagnostic journey or whose doctors were.

Furthermore, we focused solely on the direct costs from the SHI perspective. However, recent studies have highlighted the importance of additional costs associated with rare diseases such as, for example, disease-related productivity losses (e.g. absenteeism, reduced working hours) and out-of-pocket payments [12, 14, 20]. Against this background, there is scope and need for future research to quantify the indirect costs during the diagnostic process for patients with rare diseases, for example, with the help of patient and caregiver questionnaires [47, 48].

## Conclusion

This study provides comprehensive insights into the extensive diagnostic odyssey of patients with suspected but undiagnosed rare diseases. We found that the average diagnostic pathway was characterized, for example, by a higher number of hospitalizations (3.1 vs. 0.5), different diagnoses (50.0 vs. 26.4), different active substances prescribed (12.7 vs. 8.2) and number of genetic tests (14.7 vs. 0.3) compared to a matched cohort during the same observation period. Our results also provide evidence of heterogeneity in healthcare utilization during the diagnostic journey by age and sex.

This diagnostic odyssey is associated with high direct costs. In our sample, mean direct costs were around 7.6-fold higher (€26,999 vs. €3,561) than the costs that would be expected for individuals without a suspected rare disease during the same observation period. Inpatient costs accounted for the majority of total costs (62.5%). Stratification by age revealed high average annual costs for infants due to long and cost-intensive neonatal care, while total costs for older patients were comparatively high due to longer diagnostic journeys. We found no evidence for significant sex differences in diagnostic costs.

In conclusion, our results suggest that costs during the diagnostic journey contribute substantially to the overall economic burden of rare diseases. Against this background, more research on diagnostic costs and how to shorten the diagnostic journey is needed, not only for the sake of patients, but also to improve the cost-efficiency of healthcare systems.

## Electronic supplementary material

Below is the link to the electronic supplementary material.

Supplementary Material 1

## Data Availability

We used health insurance data provided by five German SHI providers (BARMER, AOK-Nordost, AOK Bayern, AOK PLUS and AOK Baden-Württemberg) but there are restrictions regarding the availability of these data to protect individuals’ privacy. Our data were used under license for the current study and are not publicly available.

## Footnotes

1 For a flowchart on the workflow used to select TNAMSE patients for this analysis, see Figure A1 in Appendix (A).

2 See Table A1 in Appendix (A) for a comparison of our study population and the excluded TNAMSE patients. The differences in age are due to the different patient structures of the specific rare disease centers and the catchment area of the data providing health insurance company (AOK).

3 In total, around 250 different rare diseases were documented for the 313 patients diagnosed with a rare disease. A total of 221 different ORPHAcodes and 29 rare diseases without existing ORPHAcode-classification were documented.

4 In a sub-analysis, Table A14 in Appendix (A) shows the results for the diagnostic pathway solely for individuals diagnosed with a rare disease. Furthermore, Table A15 in Appendix (A) reports the average diagnostic costs stratified by type of diagnosis.

5 If there were not enough suitable controls available in the three-digit ZIP-code area, controls were assigned from the two-digit ZIP-code area.

6 We further mitigated the risk of assigning individuals with undiagnosed (rare) diseases as controls by excluding individuals participating in the TNAMSE project from the pool of potential controls.

7 Of the 1,243 included TNAMSE patients, 79.5% were matched with 75 controls, 15.2% with 74 controls and for 5.3% the number of matched controls was between 35 and 73.

8 Alternative cost concepts (average costs per year observed and average costs per year insured) are provided in Tables A10-A13 in Appendix (A) as sensitivity analysis.

9 Please note that if we analyze average numbers per year of observation, instead of average total numbers, the number of hospitalizations decreased with age (Table A2, Appendix A).

10 The Tables A2-A9 in Appendix (A) provide means, standard deviations, 95%Cis and medians of all diagnostic variables stratified by sex and age for TNAMSE patients, their matched controls and in addition, for the mean differences of these two groups.

11 In our analysis, the specialist groups were derived from the 8th and 9th digit of the lifelong physician number (LANR).

12 We identified outpatient facilities using their respective pseudonym (“Betriebsstättenpseudonym”) in the outpatient claims data.

13 Additional analysis only focusing on gene panel sequencing (GOP: 11513) revealed very similar patterns but with lower average numbers.

14 See Appendix (B) for a detailed overview on the specific procedures analyzed.

15 The Tables A10-A13 in Appendix (A) provide means, standard deviations, 95%Cis and medians of all diagnostic cost components stratified by sex and age for TNAMSE patients, their matched controls and in addition, for the mean differences of these two groups.

16 The average observation period of individuals aged 1 to 17 years was slightly lower (1,232 ± 621 days) than for individuals aged 18 year or older (1,368 ± 526 days).

17 For example, one unique feature of our study is that we also included individuals seeking diagnosis for a suspected rare disease but ended up diagnosed with a common disease. Table A15 in Appendix (A) provides costs stratified by type of diagnosis. Total costs were significantly higher for individuals ending up diagnosed with a rare disease in comparison to those diagnosed with a common disease. However, validity is limited due to the low number for individuals diagnosed with a common disease (*N* = 33).

18 To address the shortcomings regarding the coding of rare diseases, an obligation to code inpatients with Alpha-ID and Orpha code (Alpha-ID-SE) was introduced in 2023 [42, 43]. Furthermore, the discussed introduction of ICD-11 would lead to more rare diseases becoming codable [44].

19 Table A1 in Appendix (A) suggests that patients in our sample might have been slightly younger than the general population of patients seeking for a diagnosis of a suspected but so far undiagnosed rare disease.
