# Supplementary material for: The cost of the diagnostic odyssey of patients with suspected rare diseases
Source: Orphanet J Rare Dis. 2025 May 10;20:222. doi: 10.1186/s13023-025-03751-y (PMC12065212; doi:10.1186/s13023-025-03751-y)
Supplement: Supplementary file 1 — Supplementary Material 1 [file 13023_2025_3751_MOESM1_ESM.docx]

**The cost of the diagnostic odyssey of**

**patients with suspected rare diseases**

Appendix – A:

Additional figures and tables

Figure A1: Flowchart of TNAMSE cohort selection


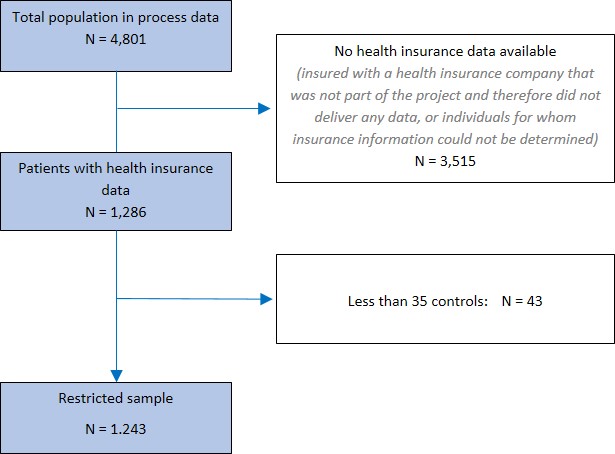


Table A1: Comparison analyzed vs. excluded TNAMSE patients

| **Indicator** | **Category** | **Analyzed**  **TNAMSE patients** | | **Excluded**  **TNAMSE patients** | |
| --- | --- | --- | --- | --- | --- |
|  |  | **N** | **(%)** | **N** | **(%)** |
| **N** |  | **1,243** |  | **3,558** |  |
| **Sex** | Men | 671 | 54 % | 1,776 | 50 % |
|  | Women | 572 | 46 % | 1,778 | 50 % |
|  | Diverse | 0 | 0 % | 2 | 0 % |
|  | N/A | 0 | 0 % | 2 | 0 % |
| **Age groups** | < 1 year | 138 | 11 % | 253 | 7 % |
|  | 1-17 years | 822 | 66 % | 1,800 | 51 % |
|  | ≥ 18 years | 283 | 23 % | 1,494 | 42 % |
|  | N/A | 0 | 0 | 11 | 0 % |
| **Diagnosis** | Common illness | 33 | 3 % | 154 | 4 % |
|  | Psychosomatic illness | 16 | 1 % | 68 | 2 % |
|  | None | 881 | 71 % | 2,726 | 77 % |
|  | Rare disease | 313 | 25 % | 609 | 17 % |
|  | N/A | 0 | 0% | 1 | 0 % |
|  |  | **Mean ± SD** | **Median** | **Mean ± SD** | **Median** |
| **Age (in years)** |  | 15 ± 18 | 7 | 23 ± 22 | 14 |

Note: Data for the analyzed TNAMSE patients was derived from SHI claims data, while we used data from project-specific patient questionnaires for excluded patients.

**Explanation:** Since specific expertise plays an important role in the diagnosis and treatment of patients with (suspected) rare diseases, the centers for rare diseases participating in TNAMSE differ substantially in terms of patient structure. The four centers for rare diseases with the lowest average age (Mean ± SD: 7.5 ± 8.5 to 17.4 ± 21.0 years) are all located in the catchment area of the health insurance (AOK) providing the data. In contrast, the three centers with the highest average age (Mean ± SD: 33.8 ± 23.2 to 40.7 ± 19.3 years) were located outside the catchment area of the AOK. As a result, the average age of patients with health insurance data was lower than that of patients without health insurance data.

Table A2: Number of hospitalizations and total duration of hospital stay [days]

|  | **Cohort** | | | | | | | | | | | | | | | | |
| --- | --- | --- | --- | --- | --- | --- | --- | --- | --- | --- | --- | --- | --- | --- | --- | --- | --- |
|  | **TNAMSE patients** | | | | | | **Control cohort** | | | | | | **Difference** | | | | |
|  | **N** | **Mean** | **± SD** | **Median** | **95% CI** | | **N** | **Mean** | **± SD** | **Median** | **95% CI** | | **Mean** | **± SD** | **Median** | **95% CI** | |
| **Total** | | | | | | |  |  |  |  |  |  |  |  |  |  |  |
| Total hospitalization | 1.243 | 3.1 | ± 5.0 | 2.0 | [2.9 ; | 3.4] | 92.078 | 0.5 | ± 1.5 | 0.0 | [0.5 ; | 0.5] | 2.6 | ± 4.9 | 1.2 | [2.4 ; | 2.9] |
| Ø hospitalizations / year | 1.243 | 1.4 | ± 3.2 | 0.7 | [1.3 ; | 1.6] | 92.078 | 0.2 | ± 0.6 | 0.0 | [0.2 ; | 0.2] | 1.2 | ± 3.2 | 0.5 | [1.1 ; | 1.4] |
| Total duration [days] | 1.243 | 21.8 | ± 47.3 | 7.0 | [19.2 ; | 24.5] | 92.078 | 2.6 | ± 13.3 | 0.0 | [2.5 ; | 2.7] | 19.2 | ± 47.0 | 4.0 | [16.6 ; | 21.8] |
| Ø duration / year | 1.243 | 12.1 | ± 33.7 | 2.2 | [10.2 ; | 14.0] | 92.078 | 1.0 | ± 6.7 | 0.0 | [1.0 ; | 1.1] | 11.0 | ± 33.4 | 1.5 | [9.2 ; | 12.9] |
| **Men** | | | | | | |  |  |  |  |  |  |  |  |  |  |  |
| Total hospitalization | 671 | 3.2 | ± 5.4 | 2.0 | [2.8 ; | 3.6] | 49.697 | 0.5 | ± 1.6 | 0.0 | [0.5 ; | 0.5] | 2.7 | ± 5.3 | 1.2 | [2.3 ; | 3.1] |
| Ø hospitalizations / year | 671 | 1.4 | ± 2.2 | 0.6 | [1.2 ; | 1.5] | 49.697 | 0.2 | ± 0.6 | 0.0 | [0.2 ; | 0.2] | 1.2 | ± 2.1 | 0.5 | [1.0 ; | 1.3] |
| Total duration [days] | 671 | 22.9 | ± 53.6 | 6.0 | [18.9 ; | 27.0] | 49.697 | 2.7 | ± 14.3 | 0.0 | [2.6 ; | 2.8] | 20.3 | ± 53.2 | 3.3 | [16.2 ; | 24.3] |
| Ø duration / year | 671 | 12.0 | ± 33.6 | 2.1 | [9.4 ; | 14.5] | 49.697 | 1.1 | ± 7.0 | 0.0 | [1.0 ; | 1.2] | 10.8 | ± 33.2 | 1.2 | [8.3 ; | 13.3] |
| **Women** | | | | | | |  |  |  |  |  |  |  |  |  |  |  |
| Total hospitalization | 572 | 3.1 | ± 4.4 | 2.0 | [2.8 ; | 3.5] | 42.381 | 0.5 | ± 1.4 | 0.0 | [0.5 ; | 0.5] | 2.6 | ± 4.3 | 1.3 | [2.3 ; | 3.0] |
| Ø hospitalizations / year | 572 | 1.5 | ± 4.2 | 0.7 | [1.2 ; | 1.9] | 42.381 | 0.2 | ± 0.5 | 0.0 | [0.2 ; | 0.2] | 1.3 | ± 4.2 | 0.5 | [1.0 ; | 1.7] |
| Total duration [days] | 572 | 20.5 | ± 38.6 | 8.0 | [17.3 ; | 23.7] | 42.381 | 2.5 | ± 12.0 | 0.0 | [2.4 ; | 2.6] | 18.0 | ± 38.5 | 4.7 | [14.8 ; | 21.2] |
| Ø duration / year | 572 | 12.3 | ± 33.9 | 2.6 | [9.5 ; | 15.1] | 42.381 | 1.0 | ± 6.3 | 0.0 | [0.9 ; | 1.1] | 11.3 | ± 33.7 | 1.8 | [8.5 ; | 14.1] |
| **Age: < 1 year** | | | | | | |  |  |  |  |  |  |  |  |  |  |  |
| Total hospitalization | 138 | 2.1 | ± 1.8 | 2.0 | [1.8 ; | 2.4] | 9.492 | 0.4 | ± 0.7 | 0.0 | [0.4 ; | 0.4] | 1.7 | ± 1.8 | 1.1 | [1.4 ; | 2.0] |
| Ø hospitalizations / year | 138 | 3.5 | ± 7.9 | 2.3 | [2.1 ; | 4.8] | 9.492 | 0.5 | ± 0.9 | 0.0 | [0.4 ; | 0.5] | 3.0 | ± 7.9 | 1.9 | [1.7 ; | 4.3] |
| Total duration [days] | 138 | 33.0 | ± 39.4 | 19.0 | [26.4 ; | 39.7] | 9.492 | 2.5 | ± 10.0 | 0.0 | [2.3 ; | 2.7] | 30.6 | ± 39.7 | 16.8 | [23.9 ; | 37.3] |
| Ø duration / year | 138 | 50.7 | ± 81.9 | 25.5 | [36.9 ; | 64.5] | 9.492 | 3.0 | ± 13.6 | 0.0 | [2.8 ; | 3.3] | 47.6 | ± 81.8 | 23.6 | [33.8 ; | 61.3] |
| **Age: 1 -17 years** | | | | | | |  |  |  |  |  |  |  |  |  |  |  |
| Total hospitalization | 822 | 3.1 | ± 5.4 | 1.0 | [2.7 ; | 3.4] | 61.416 | 0.4 | ± 1.5 | 0.0 | [0.4 ; | 0.4] | 2.6 | ± 5.3 | 1.0 | [2.3 ; | 3.0] |
| Ø hospitalizations / year | 822 | 1.2 | ± 2.1 | 0.5 | [1.1 ; | 1.3] | 61.416 | 0.1 | ± 0.5 | 0.0 | [0.1 ; | 0.1] | 1.1 | ± 2.0 | 0.4 | [0.9 ; | 1.2] |
| Total duration [days] | 822 | 19.6 | ± 49.2 | 4.0 | [16.2 ; | 22.9] | 61.416 | 2.1 | ± 11.4 | 0.0 | [2.0 ; | 2.1] | 17.5 | ± 48.8 | 2.6 | [14.2 ; | 20.9] |
| Ø duration / year | 822 | 7.6 | ± 16.8 | 1.5 | [6.5 ; | 8.8] | 61.416 | 0.7 | ± 4.5 | 0.0 | [0.6 ; | 0.7] | 6.9 | ± 16.8 | 0.8 | [5.8 ; | 8.1] |
| **Age: ≥ 18 years** | | | | | | |  |  |  |  |  |  |  |  |  |  |  |
| Total hospitalization | 283 | 3.9 | ± 4.6 | 2.0 | [3.3 ; | 4.4] | 21.170 | 0.8 | ± 1.8 | 0.0 | [0.8 ; | 0.8] | 3.1 | ± 4.6 | 1.7 | [2.6 ; | 3.6] |
| Ø hospitalizations / year | 283 | 1.1 | ± 1.2 | 0.7 | [1.0 ; | 1.3] | 21.170 | 0.2 | ± 0.6 | 0.0 | [0.2 ; | 0.2] | 0.9 | ± 1.2 | 0.5 | [0.8 ; | 1.0] |
| Total duration [days] | 283 | 22.9 | ± 44.5 | 10.0 | [17.7 ; | 28.1] | 21.170 | 4.2 | ± 18.6 | 0.0 | [4.0 ; | 4.5] | 18.7 | ± 44.3 | 6.2 | [13.5 ; | 23.8] |
| Ø duration / year | 283 | 6.4 | ± 10.6 | 2.7 | [5.1 ; | 7.6] | 21.170 | 1.2 | ± 7.3 | 0.0 | [1.1 ; | 1.3] | 5.1 | ± 10.7 | 1.7 | [3.9 ; | 6.4] |

*Notes: Means for total number of hospitalizations and total duration of hospital stays refer to all hospitalizations/days in hospital during the observation period. Furthermore, Ø hospitalizations / year and Ø duration/ year represent average values per year of observation.*

Table A3: Number of diagnoses (number of different ICD-4 digits – outpatient and inpatient)

|  |  | | | | | | | | | | | | | | | | |
| --- | --- | --- | --- | --- | --- | --- | --- | --- | --- | --- | --- | --- | --- | --- | --- | --- | --- |
|  | **TNAMSE patient** | | | | | | **Control cohort** | | | | | | **Difference** | | | | |
|  | **N** | **Mean** | **± SD** | **Median** | **95% CI** | | **N** | **Mean** | **± SD** | **Median** | **95% CI** | | **Mean** | **± SD** | **Median** | **95% CI** | |
| **Total** | | | | | | |  |  |  |  |  |  |  |  |  |  |  |
| Total number | 1,243 | 50.0 | ± 34.1 | 42.0 | [48.1 ; | 51.9] | 92,078 | 26.4 | ± 19.6 | 22.0 | [26.2 ; | 26.5] | 23.7 | ± 29.0 | 16.8 | [22.1 ; | 25.4] |
| **Sex: male** | | | | | | |  |  |  |  |  |  |  |  |  |  |  |
| Total number | 671 | 47.0 | ± 29.7 | 41.0 | [44.7 ; | 49.2] | 49,697 | 25.6 | ± 18.4 | 22.0 | [25.5 ; | 25.8] | 21.5 | ± 26.0 | 16.1 | [19.5 ; | 23.4] |
| **Sex: female** | | | | | | |  |  |  |  |  |  |  |  |  |  |  |
| Total number | 572 | 53.5 | ± 38.4 | 43.5 | [50.4 ; | 56.7] | 42,381 | 27.3 | ± 20.9 | 23.0 | [27.1 ; | 27.5] | 26.4 | ± 32.0 | 18.0 | [23.8 ; | 29.0] |
| **Age: < 1 year** | | | | | | |  |  |  |  |  |  |  |  |  |  |  |
| Total number | 138 | 26.4 | ± 15.1 | 25.0 | [23.9 ; | 29.0] | 9,492 | 13.8 | ± 8.7 | 12.5 | [13.6 ; | 13.9] | 13.0 | ± 14.4 | 12.0 | [10.6 ; | 15.4] |
| **Age: 1 -17 years** | | | | | | |  |  |  |  |  |  |  |  |  |  |  |
| Total number | 822 | 46.1 | ± 27.3 | 41.0 | [44.2 ; | 48.0] | 61,416 | 26.5 | ± 17.8 | 24.0 | [26.4 ; | 26.7] | 19.5 | ± 23.1 | 14.6 | [17.9 ; | 21.1] |
| **Age: ≥ 18 years** | | | | | | |  |  |  |  |  |  |  |  |  |  |  |
| Total number | 283 | 72.8 | ± 44.8 | 64.0 | [67.5 ; | 78.0] | 21,170 | 31.5 | ± 24.9 | 26.0 | [31.2 ; | 31.9] | 41.2 | ± 40.4 | 33.5 | [36.5 ; | 46.0] |

*Notes: The number of diagnoses were derived from analyzing all different ICD-10 codes documented for both inpatient and outpatient care during the observation period. Means for total number of diagnoses refer to all diagnoses during the observation period.*

Table A4: Outpatient treatment: Number of specialist groups and operating facilities

|  | **Cohort** | | | | | | | | | | | | | | | | |
| --- | --- | --- | --- | --- | --- | --- | --- | --- | --- | --- | --- | --- | --- | --- | --- | --- | --- |
|  | **TNAMSE patient** | | | | | | **Control cohort** | | | | | | **Difference** | | | | |
|  | **N** | **Mean** | **± SD** | **Median** | **95% CI** | | **N** | **Mean** | **± SD** | **Median** | **95% CI** | | **Mean** | **± SD** | **Median** | **95% CI** | |
| **Total** | | | | | | |  |  |  |  |  |  |  |  |  |  |  |
| Number of specialists | 1,243 | 7.3 | ± 5.1 | 6.0 | [7.0 ; | 7.5] | 92,078 | 4.3 | ± 3.2 | 4.0 | [4.3 ; | 4.4] | 2.9 | ± 3.8 | 2.3 | [2.7 ; | 3.1] |
| Ø number / year | 1,243 | 3.0 | ± 3.1 | 2.1 | [2.8 ; | 3.1] | 92,078 | 1.7 | ± 2.0 | 1.3 | [1.7 ; | 1.7] | 1.2 | ± 2.4 | 0.8 | [1.1 ; | 1.4] |
| Number of operating sites | 1,243 | 12.5 | ± 11.5 | 9.0 | [11.8 ; | 13.1] | 92,078 | 6.6 | ± 5.4 | 5.0 | [6.6 ; | 6.6] | 5.9 | ± 9.6 | 3.6 | [5.4 ; | 6.5] |
| Ø number / year | 1,243 | 5.2 | ± 9.6 | 3.4 | [4.7 ; | 5.7] | 92,078 | 2.7 | ± 6.0 | 2.0 | [2.6 ; | 2.7] | 2.5 | ± 5.8 | 1.3 | [2.2 ; | 2.8] |
| **Sex: male** | | | | | | |  |  |  |  |  |  |  |  |  |  |  |
| Number of specialists | 671 | 6.6 | ± 4.1 | 6.0 | [6.3 ; | 6.9] | 49,697 | 4.0 | ± 2.8 | 3.0 | [4.0 ; | 4.1] | 2.6 | ± 3.3 | 2.1 | [2.3 ; | 2.8] |
| Ø number / year | 671 | 2.6 | ± 2.7 | 1.9 | [2.4 ; | 2.8] | 49,697 | 1.6 | ± 1.7 | 1.2 | [1.6 ; | 1.6] | 1.0 | ± 2.2 | 0.7 | [0.9 ; | 1.2] |
| Number of operating sites | 671 | 10.9 | ± 8.0 | 9.0 | [10.3 ; | 11.5] | 49,697 | 6.0 | ± 4.5 | 5.0 | [6.0 ; | 6.1] | 4.9 | ± 7.0 | 3.4 | [4.3 ; | 5.4] |
| Ø number / year | 671 | 4.3 | ± 4.8 | 3.0 | [3.9 ; | 4.6] | 49,697 | 2.3 | ± 2.8 | 1.8 | [2.3 ; | 2.3] | 2.0 | ± 3.9 | 1.1 | [1.7 ; | 2.3] |
| **Sex: female** | | | | | | |  |  |  |  |  |  |  |  |  |  |  |
| Number of specialists | 572 | 8.0 | ± 5.9 | 7.0 | [7.5 ; | 8.5] | 42,381 | 4.7 | ± 3.6 | 4.0 | [4.7 ; | 4.7] | 3.4 | ± 4.2 | 2.5 | [3.0 ; | 3.7] |
| Ø number / year | 572 | 3.3 | ± 3.4 | 2.5 | [3.1 ; | 3.6] | 42,381 | 1.9 | ± 2.3 | 1.4 | [1.9 ; | 1.9] | 1.4 | ± 2.6 | 0.9 | [1.2 ; | 1.6] |
| Number of operating sites | 572 | 14.4 | ± 14.4 | 10.0 | [13.2 ; | 15.5] | 42,381 | 7.2 | ± 6.3 | 5.0 | [7.2 ; | 7.3] | 7.2 | ± 11.8 | 4.2 | [6.2 ; | 8.2] |
| Ø number / year | 572 | 6.3 | ± 13.2 | 4.0 | [5.2 ; | 7.4] | 42,381 | 3.1 | ± 8.3 | 2.1 | [3.0 ; | 3.2] | 3.2 | ± 7.3 | 1.6 | [2.6 ; | 3.8] |
| **Age: < 1 year** | | | | | | |  |  |  |  |  |  |  |  |  |  |  |
| Number of specialists | 138 | 2.1 | ± 2.0 | 2.0 | [1.8 ; | 2.5] | 9,492 | 1.7 | ± 1.2 | 2.0 | [1.7 ; | 1.7] | 0.5 | ± 1.8 | 0.0 | [0.2 ; | 0.8] |
| Ø number / year | 138 | 2.7 | ± 3.0 | 2.0 | [2.2 ; | 3.2] | 9,492 | 2.2 | ± 3.1 | 1.9 | [2.2 ; | 2.3] | 0.5 | ± 2.8 | 0.0 | [0.0 ; | 0.9] |
| Number of operating sites | 138 | 3.4 | ± 2.7 | 3.0 | [2.9 ; | 3.8] | 9,492 | 2.4 | ± 1.6 | 2.0 | [2.4 ; | 2.5] | 1.0 | ± 2.5 | 0.4 | [0.6 ; | 1.4] |
| Ø number / year | 138 | 6.4 | ± 23.3 | 3.3 | [2.5 ; | 10.3] | 9,492 | 4.3 | ± 16.4 | 2.4 | [4.0 ; | 4.7] | 2.1 | ± 10.7 | 0.4 | [0.3 ; | 3.9] |
| **Age: 1 -17 years** | | | | | | |  |  |  |  |  |  |  |  |  |  |  |
| Number of specialists | 822 | 6.2 | ± 3.1 | 6.0 | [6.0 ; | 6.4] | 61,416 | 3.9 | ± 2.6 | 3.0 | [3.9 ; | 4.0] | 2.2 | ± 2.7 | 2.0 | [2.0 ; | 2.4] |
| Ø number / year | 822 | 2.5 | ± 2.8 | 1.8 | [2.3 ; | 2.7] | 61,416 | 1.5 | ± 1.6 | 1.2 | [1.5 ; | 1.5] | 1.0 | ± 2.2 | 0.6 | [0.9 ; | 1.2] |
| Number of operating sites | 822 | 10.0 | ± 5.9 | 9.0 | [9.6 ; | 10.4] | 61,416 | 6.0 | ± 4.2 | 5.0 | [5.9 ; | 6.0] | 4.0 | ± 5.2 | 3.2 | [3.7 ; | 4.4] |
| Ø number / year | 822 | 4.0 | ± 4.6 | 2.9 | [3.7 ; | 4.4] | 61,416 | 2.2 | ± 2.3 | 1.7 | [2.2 ; | 2.2] | 1.8 | ± 3.6 | 1.0 | [1.6 ; | 2.1] |
| **Age: ≥ 18 years** | | | | | | |  |  |  |  |  |  |  |  |  |  |  |
| Number of specialists | 283 | 12.9 | ± 5.9 | 12.0 | [12.2 ; | 13.6] | 21,170 | 6.7 | ± 4.1 | 6.0 | [6.6 ; | 6.7] | 6.2 | ± 5.1 | 5.7 | [5.6 ; | 6.8] |
| Ø number / year | 283 | 4.3 | ± 3.5 | 3.5 | [3.9 ; | 4.7] | 21,170 | 2.2 | ± 2.3 | 1.7 | [2.1 ; | 2.2] | 2.1 | ± 2.5 | 1.6 | [1.8 ; | 2.4] |
| Number of operating sites | 283 | 24.1 | ± 16.9 | 20.0 | [22.1 ; | 26.0] | 21,170 | 10.3 | ± 7.2 | 9.0 | [10.2 ; | 10.4] | 13.8 | ± 15.5 | 10.2 | [12.0 ; | 15.6] |
| Ø number / year | 283 | 8.0 | ± 8.5 | 5.8 | [7.0 ; | 9.0] | 21,170 | 3.3 | ± 4.2 | 2.6 | [3.2 ; | 3.4] | 4.7 | ± 6.8 | 3.0 | [3.9 ; | 5.5] |

*Notes: Outpatient specialist groups were derived from the 8th and 9th digit of the lifelong physician number (LANR). Outpatient facilities were identified using their respective pseudonym (“Betriebsstättenpseudonym”) in the outpatient claims data. Means for total number of specialist groups and operating facilities refer to all specialist groups and operating facilities visited during the observation period. Furthermore, Ø number/year represent the respective average values per year of observation.*

Table A5: Number of different active substances prescribed

|  | **Cohort** | | | | | | | | | | | | | | | | |
| --- | --- | --- | --- | --- | --- | --- | --- | --- | --- | --- | --- | --- | --- | --- | --- | --- | --- |
|  | **TNAMSE patient** | | | | | | **Control cohort** | | | | | | **Difference** | | | | |
|  | **N** | **Mean** | **± SD** | **Median** | **95% CI** | | **N** | **Mean** | **± SD** | **Median** | **95% CI** | | **Mean** | **± SD** | **Median** | **95% CI** | |
| **Total** | | | | | | |  |  |  |  |  |  |  |  |  |  |  |
| Total number | 1,243 | 12.7 | ± 9.8 | 11.0 | [12.2 ; | 13.3] | 92,078 | 8.2 | ± 7.0 | 7.0 | [8.2 ; | 8.3] | 4.6 | ± 8.5 | 2.7 | [4.1 ; | 5.1] |
| Ø number / year | 1,243 | 4.7 | ± 4.3 | 3.8 | [4.5 ; | 5.0] | 92,078 | 3.1 | ± 3.3 | 2.4 | [3.1 ; | 3.1] | 1.6 | ± 3.9 | 1.0 | [1.4 ; | 1.8] |
| **Sex: male** | | | | | | |  |  |  |  |  |  |  |  |  |  |  |
| Total number | 671 | 12.7 | ± 9.6 | 11.0 | [12.0 ; | 13.5] | 49,697 | 8.6 | ± 7.3 | 7.0 | [8.5 ; | 8.6] | 4.2 | ± 8.2 | 2.4 | [3.6 ; | 4.8] |
| Ø number / year | 671 | 4.5 | ± 3.9 | 3.8 | [4.2 ; | 4.8] | 49,697 | 3.2 | ± 3.3 | 2.5 | [3.1 ; | 3.2] | 1.3 | ± 3.4 | 0.9 | [1.1 ; | 1.6] |
| **Sex: female** | | | | | | |  |  |  |  |  |  |  |  |  |  |  |
| Total number | 572 | 12.8 | ± 10.0 | 11.0 | [12.0 ; | 13.6] | 42,381 | 7.8 | ± 6.7 | 6.0 | [7.8 ; | 7.9] | 5.8 | ± 8.9 | 4.0 | [5.1 ; | 6.5] |
| Ø number / year | 572 | 5.0 | ± 4.7 | 4.0 | [4.6 ; | 5.4] | 42,381 | 3.1 | ± 3.4 | 2.3 | [3.0 ; | 3.1] | 1.9 | ± 4.3 | 1.1 | [1.6 ; | 2.3] |

| **Age: < 1 year** | | | | | | |  |  |  |  |  |  |  |  |  |  |  |
| --- | --- | --- | --- | --- | --- | --- | --- | --- | --- | --- | --- | --- | --- | --- | --- | --- | --- |
| Total number | 138 | 4.2 | ± 4.6 | 3.0 | [3.5 ; | 5.0] | 9,492 | 3.5 | ± 3.2 | 3.0 | [3.5 ; | 3.6] | 0.8 | ± 4.0 | 0.0 | [0.2 ; | 1.5] |
| Ø number / year | 138 | 5.1 | ± 5.9 | 3.5 | [4.1 ; | 6.1] | 9,492 | 4.2 | ± 4.8 | 3.3 | [4.1 ; | 4.3] | 0.9 | ± 5.5 | -0.3 | [0.0 ; | 1.9] |
| **Age: 1 -17 years** | | | | | | |  |  |  |  |  |  |  |  |  |  |  |
| Total number | 822 | 13.6 | ± 9.4 | 12.0 | [12.9 ; | 14.2] | 61,416 | 9.5 | ± 7.3 | 8.0 | [9.5 ; | 9.6] | 4.0 | ± 8.1 | 2.3 | [3.5 ; | 4.6] |
| Ø number / year | 822 | 4.8 | ± 4.1 | 3.9 | [4.5 ; | 5.1] | 61,416 | 3.3 | ± 3.2 | 2.7 | [3.3 ; | 3.3] | 1.4 | ± 3.7 | 0.8 | [1.2 ; | 1.7] |
| **Age: ≥ 18 years** | | | | | | |  |  |  |  |  |  |  |  |  |  |  |
| Total number | 283 | 14.5 | ± 10.6 | 13.0 | [13.3 ; | 15.8] | 21,170 | 6.5 | ± 6.1 | 5.0 | [6.4 ; | 6.6] | 9.0 | ± 9.9 | 7.0 | [7.9 ; | 10.2] |
| Ø number / year | 283 | 4.4 | ± 3.7 | 3.6 | [4.0 ; | 4.8] | 21,170 | 2.0 | ± 2.6 | 1.4 | [2.0 ; | 2.0] | 2.4 | ± 3.4 | 1.9 | [2.0 ; | 2.8] |

*Notes: The number of different active substances prescribed is analyzed using claims data on 5-digit ATC codes. Means for number of different active substances prescribed refer to all active substances prescribed during the observation period. Furthermore, Ø number/year represent the respective average values per year of observation.*

Table A6: Number of imaging procedures (CT, MRI, PET, SPECT, Scintigraphy)

|  | **Cohort** | | | | | | | | | | | | | | | | |
| --- | --- | --- | --- | --- | --- | --- | --- | --- | --- | --- | --- | --- | --- | --- | --- | --- | --- |
|  | **TNAMSE patient** | | | | | | **Control cohort** | | | | | | **Difference** | | | | |
|  | **N** | **Mean** | **± SD** | **Median** | **95% CI** | | **N** | **Mean** | **± SD** | **Median** | **95% CI** | | **Mean** | **± SD** | **Median** | **95% CI** | |
| **Total** | | | | | | |  |  |  |  |  |  |  |  |  |  |  |
| **Total** number | 1,243 | 2.1 | ± 4.3 | 1.0 | [1.8 ; | 2.3] | 92,078 | 0.3 | ± 1.4 | 0.0 | [0.3 ; | 0.3] | 1.8 | ± 4.1 | 0.8 | [1.6 ; | 2.0] |
| Ø number / year | 1,243 | 1.0 | ± 2.7 | 0.2 | [0.8 ; | 1.1] | 92,078 | 0.1 | ± 0.4 | 0.0 | [0.1 ; | 0.1] | 0.9 | ± 2.7 | 0.1 | [0.7 ; | 1.0] |
| **CT** total | 1,243 | 0.5 | ± 1.7 | 0.0 | [0.4 ; | 0.6] | 92,078 | 0.1 | ± 0.7 | 0.0 | [0.1 ; | 0.1] | 0.4 | ± 1.6 | 0.0 | [0.3 ; | 0.5] |
| Ø number / year | 1,243 | 0.2 | ± 0.6 | 0.0 | [0.2 ; | 0.2] | 92,078 | 0.0 | ± 0.2 | 0.0 | [0.0 ; | 0.0] | 0.2 | ± 0.6 | 0.0 | [0.1 ; | 0.2] |
| **MRI** total | 1,243 | 1.5 | ± 3.2 | 0.0 | [1.3 ; | 1.7] | 92,078 | 0.2 | ± 1.0 | 0.0 | [0.2 ; | 0.2] | 1.3 | ± 3.1 | 0.0 | [1.2 ; | 1.5] |
| Ø number / year | 1,243 | 0.7 | ± 2.4 | 0.0 | [0.6 ; | 0.9] | 92,078 | 0.1 | ± 0.3 | 0.0 | [0.1 ; | 0.1] | 0.7 | ± 2.4 | 0.0 | [0.6 ; | 0.8] |
| **PET** total | 1,243 | 0.0 | ± 0.1 | 0.0 | [0.0 ; | 0.0] | 92,078 | 0.0 | ± 0.0 | 0.0 | [0.0 ; | 0.0] | 0.0 | ± 0.1 | 0.0 | [0.0 ; | 0.0] |
| Ø number / year | 1,243 | 0.0 | ± 0.1 | 0.0 | [0.0 ; | 0.0] | 92,078 | 0.0 | ± 0.0 | 0.0 | [0.0 ; | 0.0] | 0.0 | ± 0.1 | 0.0 | [0.0 ; | 0.0] |
| **SPECT** total | 1,243 | 0.0 | ± 0.1 | 0.0 | [0.0 ; | 0.0] | 92,078 | 0.0 | ± 0.1 | 0.0 | [0.0 ; | 0.0] | 0.0 | ± 0.1 | 0.0 | [0.0 ; | 0.0] |
| Ø number / year | 1,243 | 0.0 | ± 0.0 | 0.0 | [0.0 ; | 0.0] | 92,078 | 0.0 | ± 0.0 | 0.0 | [0.0 ; | 0.0] | 0.0 | ± 0.0 | 0.0 | [0.0 ; | 0.0] |
| **Scintigraphy** total | 1,243 | 0.1 | ± 0.3 | 0.0 | [0.0 ; | 0.1] | 92,078 | 0.0 | ± 0.2 | 0.0 | [0.0 ; | 0.0] | 0.1 | ± 0.3 | 0.0 | [0.0 ; | 0.1] |
| Ø number / year | 1,243 | 0.0 | ± 0.2 | 0.0 | [0.0 ; | 0.0] | 92,078 | 0.0 | ± 0.1 | 0.0 | [0.0 ; | 0.0] | 0.0 | ± 0.2 | 0.0 | [0.0 ; | 0.0] |
| **Sex: male** | | | | | | |  |  |  |  |  |  |  |  |  |  |  |
| Total number | 671 | 1.8 | ± 4.5 | 0.0 | [1.4 ; | 2.1] | 49,697 | 0.2 | ± 1.3 | 0.0 | [0.2 ; | 0.2] | 1.5 | ± 4.4 | 0.0 | [1.2 ; | 1.9] |
| Ø number / year | 671 | 0.7 | ± 1.9 | 0.0 | [0.6 ; | 0.9] | 49,697 | 0.1 | ± 0.4 | 0.0 | [0.1 ; | 0.1] | 0.7 | ± 1.9 | 0.0 | [0.5 ; | 0.8] |
| **CT** total | 671 | 0.4 | ± 1.6 | 0.0 | [0.3 ; | 0.6] | 49,697 | 0.1 | ± 0.6 | 0.0 | [0.1 ; | 0.1] | 0.4 | ± 1.6 | 0.0 | [0.3 ; | 0.5] |
| Ø number / year | 671 | 0.2 | ± 0.6 | 0.0 | [0.1 ; | 0.2] | 49,697 | 0.0 | ± 0.2 | 0.0 | [0.0 ; | 0.0] | 0.2 | ± 0.6 | 0.0 | [0.1 ; | 0.2] |
| **MRI** total | 671 | 1.3 | ± 3.4 | 0.0 | [1.0 ; | 1.5] | 49,697 | 0.2 | ± 0.8 | 0.0 | [0.1 ; | 0.2] | 1.1 | ± 3.3 | 0.0 | [0.9 ; | 1.4] |
| Ø number / year | 671 | 0.6 | ± 1.7 | 0.0 | [0.4 ; | 0.7] | 49,697 | 0.0 | ± 0.3 | 0.0 | [0.0 ; | 0.0] | 0.5 | ± 1.7 | 0.0 | [0.4 ; | 0.6] |
| **PET** total | 671 | 0.0 | ± 0.1 | 0.0 | [0.0 ; | 0.0] | 49,697 | 0.0 | ± 0.0 | 0.0 | [0.0 ; | 0.0] | 0.0 | ± 0.1 | 0.0 | [0.0 ; | 0.0] |
| Ø number / year | 671 | 0.0 | ± 0.0 | 0.0 | [0.0 ; | 0.0] | 49,697 | 0.0 | ± 0.0 | 0.0 | [0.0 ; | 0.0] | 0.0 | ± 0.0 | 0.0 | [0.0 ; | 0.0] |
| **SPCT** total | 671 | 0.0 | ± 0.1 | 0.0 | [0.0 ; | 0.0] | 49,697 | 0.0 | ± 0.1 | 0.0 | [0.0 ; | 0.0] | 0.0 | ± 0.1 | 0.0 | [0.0 ; | 0.0] |
| Ø number / year | 671 | 0.0 | ± 0.0 | 0.0 | [0.0 ; | 0.0] | 49,697 | 0.0 | ± 0.0 | 0.0 | [0.0 ; | 0.0] | 0.0 | ± 0.0 | 0.0 | [0.0 ; | 0.0] |
| **Scintigraphy** total | 671 | 0.0 | ± 0.2 | 0.0 | [0.0 ; | 0.1] | 49,697 | 0.0 | ± 0.1 | 0.0 | [0.0 ; | 0.0] | 0.0 | ± 0.2 | 0.0 | [0.0 ; | 0.1] |
| Ø number / year | 671 | 0.0 | ± 0.1 | 0.0 | [0.0 ; | 0.0] | 49,697 | 0.0 | ± 0.1 | 0.0 | [0.0 ; | 0.0] | 0.0 | ± 0.1 | 0.0 | [0.0 ; | 0.0] |

**For a continuation of Table A6, see next page.**

|  | **Cohort** | | | | | | | | | | | | | | | | |
| --- | --- | --- | --- | --- | --- | --- | --- | --- | --- | --- | --- | --- | --- | --- | --- | --- | --- |
|  | **TNAMSE patient** | | | | | | **Control cohort** | | | | | | **Difference** | | | | |
|  | **N** | **Mean** | **± SD** | **Median** | **95% CI** | | **N** | **Mean** | **± SD** | **Median** | **95% CI** | | **Mean** | **± SD** | **Median** | **95% CI** | |
| **Sex: female** | | | | | | |  |  |  |  |  |  |  |  |  |  |  |
| Total number | 572 | 2.5 | ± 4.1 | 1.0 | [2.1 ; | 2.8] | 42,381 | 0.4 | ± 1.6 | 0.0 | [0.4 ; | 0.4] | 2.1 | ± 3.8 | 0.9 | [1.8 ; | 2.4] |
| Ø number / year | 572 | 1.2 | ± 3.4 | 0.3 | [0.9 ; | 1.5] | 42,381 | 0.1 | ± 0.5 | 0.0 | [0.1 ; | 0.1] | 1.1 | ± 3.4 | 0.2 | [0.8 ; | 1.4] |
| **CT** total | 572 | 0.6 | ± 1.8 | 0.0 | [0.4 ; | 0.7] | 42,381 | 0.1 | ± 0.8 | 0.0 | [0.1 ; | 0.1] | 0.4 | ± 1.7 | 0.0 | [0.3 ; | 0.6] |
| Ø number / year | 572 | 0.2 | ± 0.7 | 0.0 | [0.1 ; | 0.3] | 42,381 | 0.0 | ± 0.3 | 0.0 | [0.0 ; | 0.0] | 0.2 | ± 0.6 | 0.0 | [0.1 ; | 0.2] |
| **MRI** total | 572 | 1.8 | ± 3.0 | 1.0 | [1.6 ; | 2.1] | 42,381 | 0.3 | ± 1.1 | 0.0 | [0.2 ; | 0.3] | 1.6 | ± 2.8 | 0.5 | [1.3 ; | 1.8] |
| Ø number / year | 572 | 1.0 | ± 3.1 | 0.2 | [0.7 ; | 1.2] | 42,381 | 0.1 | ± 0.3 | 0.0 | [0.1 ; | 0.1] | 0.9 | ± 3.1 | 0.1 | [0.6 ; | 1.2] |
| **PET** total | 572 | 0.0 | ± 0.1 | 0.0 | [0.0 ; | 0.0] | 42,381 | 0.0 | ± 0.0 | 0.0 | [0.0 ; | 0.0] | 0.0 | ± 0.1 | 0.0 | [0.0 ; | 0.0] |
| Ø number / year | 572 | 0.0 | ± 0.1 | 0.0 | [0.0 ; | 0.0] | 42,381 | 0.0 | ± 0.0 | 0.0 | [0.0 ; | 0.0] | 0.0 | ± 0.1 | 0.0 | [0.0 ; | 0.0] |
| **SPECT** total | 572 | 0.0 | ± 0.1 | 0.0 | [0.0 ; | 0.0] | 42,381 | 0.0 | ± 0.0 | 0.0 | [0.0 ; | 0.0] | 0.0 | ± 0.1 | 0.0 | [0.0 ; | 0.0] |
| Ø number / year | 572 | 0.0 | ± 0.0 | 0.0 | [0.0 ; | 0.0] | 42,381 | 0.0 | ± 0.0 | 0.0 | [0.0 ; | 0.0] | 0.0 | ± 0.0 | 0.0 | [0.0 ; | 0.0] |
| **Scintigraphy** total | 572 | 0.1 | ± 0.4 | 0.0 | [0.1 ; | 0.1] | 42,381 | 0.0 | ± 0.2 | 0.0 | [0.0 ; | 0.0] | 0.1 | ± 0.4 | 0.0 | [0.0 ; | 0.1] |
| Ø number / year | 572 | 0.0 | ± 0.2 | 0.0 | [0.0 ; | 0.1] | 42,381 | 0.0 | ± 0.1 | 0.0 | [0.0 ; | 0.0] | 0.0 | ± 0.2 | 0.0 | [0.0 ; | 0.1] |
| **Age: < 1 year** | | | | | | |  |  |  |  |  |  |  |  |  |  |  |
| Total number | 138 | 0.9 | ± 1.3 | 0.5 | [0.6 ; | 1.1] | 9,492 | 0.0 | ± 0.2 | 0.0 | [0.0 ; | 0.0] | 0.8 | ± 1.3 | 0.5 | [0.6 ; | 1.1] |
| Ø number / year | 138 | 1.2 | ± 2.3 | 0.3 | [0.9 ; | 1.6] | 9,492 | 0.0 | ± 0.2 | 0.0 | [0.0 ; | 0.0] | 1.2 | ± 2.3 | 0.3 | [0.8 ; | 1.6] |
| **CT** total | 138 | 0.1 | ± 0.5 | 0.0 | [0.1 ; | 0.2] | 9,492 | 0.0 | ± 0.1 | 0.0 | [0.0 ; | 0.0] | 0.1 | ± 0.5 | 0.0 | [0.1 ; | 0.2] |
| Ø number / year | 138 | 0.2 | ± 0.5 | 0.0 | [0.1 ; | 0.2] | 9,492 | 0.0 | ± 0.1 | 0.0 | [0.0 ; | 0.0] | 0.2 | ± 0.5 | 0.0 | [0.1 ; | 0.2] |
| **MR**I total | 138 | 0.7 | ± 1.2 | 0.0 | [0.5 ; | 0.9] | 9,492 | 0.0 | ± 0.1 | 0.0 | [0.0 ; | 0.0] | 0.7 | ± 1.2 | 0.0 | [0.5 ; | 0.9] |
| Ø number / year | 138 | 1.1 | ± 2.3 | 0.0 | [0.7 ; | 1.5] | 9,492 | 0.0 | ± 0.2 | 0.0 | [0.0 ; | 0.0] | 1.1 | ± 2.3 | 0.0 | [0.7 ; | 1.5] |
| **PET** total | 138 | 0.0 | ± 0.1 | 0.0 | [-0.0 ; | 0.0] | 9,492 | 0.0 | ± 0.0 | 0.0 | [0.0 ; | 0.0] | 0.0 | ± 0.1 | 0.0 | [-0.0 ; | 0.0] |
| Ø number / year | 138 | 0.0 | ± 0.1 | 0.0 | [-0.0 ; | 0.0] | 9,492 | 0.0 | ± 0.0 | 0.0 | [0.0 ; | 0.0] | 0.0 | ± 0.1 | 0.0 | [-0.0 ; | 0.0] |
| **SPECT** total | 138 | 0.0 | ± 0.0 | 0.0 | [0.0 ; | 0.0] | 9,492 | 0.0 | ± 0.0 | 0.0 | [0.0 ; | 0.0] | 0.0 | ± 0.0 | 0.0 | [0.0 ; | 0.0] |
| Ø number / year | 138 | 0.0 | ± 0.0 | 0.0 | [0.0 ; | 0.0] | 9,492 | 0.0 | ± 0.0 | 0.0 | [0.0 ; | 0.0] | 0.0 | ± 0.0 | 0.0 | [0.0 ; | 0.0] |
| **Scintigraphy** total | 138 | 0.0 | ± 0.1 | 0.0 | [-0.0 ; | 0.0] | 9,492 | 0.0 | ± 0.0 | 0.0 | [0.0 ; | 0.0] | 0.0 | ± 0.1 | 0.0 | [-0.0 ; | 0.0] |
| Ø number / year | 138 | 0.0 | ± 0.1 | 0.0 | [-0.0 ; | 0.0] | 9,492 | 0.0 | ± 0.0 | 0.0 | [0.0 ; | 0.0] | 0.0 | ± 0.1 | 0.0 | [-0.0 ; | 0.0] |

**For a continuation of Table A6, see next page.**

| **Age: 1 -17 years** | | | | | | |  |  |  |  |  |  |  |  |  |  |  |
| --- | --- | --- | --- | --- | --- | --- | --- | --- | --- | --- | --- | --- | --- | --- | --- | --- | --- |
| Total number | 822 | 1.2 | ± 3.1 | 0.0 | [1.0 ; | 1.4] | 61,416 | 0.1 | ± 0.9 | 0.0 | [0.1 ; | 0.1] | 1.1 | ± 3.1 | 0.0 | [0.9 ; | 1.3] |
| Ø number / year | 822 | 0.6 | ± 2.7 | 0.0 | [0.5 ; | 0.8] | 61,416 | 0.0 | ± 0.3 | 0.0 | [0.0 ; | 0.0] | 0.6 | ± 2.7 | 0.0 | [0.4 ; | 0.8] |
| **CT** total | 822 | 0.1 | ± 0.7 | 0.0 | [0.1 ; | 0.2] | 61,416 | 0.0 | ± 0.2 | 0.0 | [0.0 ; | 0.0] | 0.1 | ± 0.7 | 0.0 | [0.1 ; | 0.2] |
| Ø number / year | 822 | 0.1 | ± 0.4 | 0.0 | [0.0 ; | 0.1] | 61,416 | 0.0 | ± 0.1 | 0.0 | [0.0 ; | 0.0] | 0.1 | ± 0.4 | 0.0 | [0.0 ; | 0.1] |
| **MRI** total | 822 | 1.0 | ± 2.7 | 0.0 | [0.8 ; | 1.2] | 61,416 | 0.1 | ± 0.7 | 0.0 | [0.1 ; | 0.1] | 0.9 | ± 2.7 | 0.0 | [0.8 ; | 1.1] |
| Ø number / year | 822 | 0.6 | ± 2.6 | 0.0 | [0.4 ; | 0.7] | 61,416 | 0.0 | ± 0.2 | 0.0 | [0.0 ; | 0.0] | 0.5 | ± 2.6 | 0.0 | [0.4 ; | 0.7] |
| **PET** total | 822 | 0.0 | ± 0.1 | 0.0 | [0.0 ; | 0.0] | 61,416 | 0.0 | ± 0.0 | 0.0 | [0.0 ; | 0.0] | 0.0 | ± 0.1 | 0.0 | [0.0 ; | 0.0] |
| Ø number / year | 822 | 0.0 | ± 0.0 | 0.0 | [0.0 ; | 0.0] | 61,416 | 0.0 | ± 0.0 | 0.0 | [0.0 ; | 0.0] | 0.0 | ± 0.0 | 0.0 | [0.0 ; | 0.0] |
| **SPECT** total | 822 | 0.0 | ± 0.0 | 0.0 | [0.0 ; | 0.0] | 61,416 | 0.0 | ± 0.0 | 0.0 | [0.0 ; | 0.0] | 0.0 | ± 0.0 | 0.0 | [0.0 ; | 0.0] |
| Ø number / year | 822 | 0.0 | ± 0.0 | 0.0 | [0.0 ; | 0.0] | 61,416 | 0.0 | ± 0.0 | 0.0 | [0.0 ; | 0.0] | 0.0 | ± 0.0 | 0.0 | [0.0 ; | 0.0] |
| **Scintigraphy** total | 822 | 0.0 | ± 0.1 | 0.0 | [0.0 ; | 0.0] | 61,416 | 0.0 | ± 0.1 | 0.0 | [0.0 ; | 0.0] | 0.0 | ± 0.1 | 0.0 | [0.0 ; | 0.0] |
| Ø number / year | 822 | 0.0 | ± 0.1 | 0.0 | [0.0 ; | 0.0] | 61,416 | 0.0 | ± 0.0 | 0.0 | [0.0 ; | 0.0] | 0.0 | ± 0.1 | 0.0 | [0.0 ; | 0.0] |
| **Age: ≥ 18 years** | | | | | | |  |  |  |  |  |  |  |  |  |  |  |
| Total number | 283 | 5.3 | ± 6.3 | 4.0 | [4.6 ; | 6.1] | 21,170 | 1.0 | ± 2.4 | 0.0 | [1.0 ; | 1.1] | 4.3 | ± 6.2 | 2.7 | [3.6 ; | 5.0] |
| Ø number / year | 283 | 1.7 | ± 2.7 | 1.0 | [1.4 ; | 2.1] | 21,170 | 0.3 | ± 0.7 | 0.0 | [0.3 ; | 0.3] | 1.5 | ± 2.7 | 0.8 | [1.1 ; | 1.8] |
| **CT** total | 283 | 1.7 | ± 3.0 | 0.0 | [1.3 ; | 2.0] | 21,170 | 0.4 | ± 1.5 | 0.0 | [0.4 ; | 0.4] | 1.3 | ± 2.9 | 0.0 | [1.0 ; | 1.7] |
| Ø number / year | 283 | 0.5 | ± 1.0 | 0.0 | [0.4 ; | 0.7] | 21,170 | 0.1 | ± 0.5 | 0.0 | [0.1 ; | 0.1] | 0.4 | ± 1.0 | 0.0 | [0.3 ; | 0.6] |
| **MRI** total | 283 | 3.4 | ± 4.4 | 2.0 | [2.9 ; | 3.9] | 21,170 | 0.6 | ± 1.5 | 0.0 | [0.6 ; | 0.6] | 2.8 | ± 4.3 | 1.6 | [2.3 ; | 3.3] |
| Ø number / year | 283 | 1.1 | ± 2.0 | 0.6 | [0.9 ; | 1.3] | 21,170 | 0.2 | ± 0.4 | 0.0 | [0.2 ; | 0.2] | 0.9 | ± 1.9 | 0.4 | [0.7 ; | 1.2] |
| **PET** total | 283 | 0.0 | ± 0.2 | 0.0 | [0.0 ; | 0.1] | 21,170 | 0.0 | ± 0.1 | 0.0 | [0.0 ; | 0.0] | 0.0 | ± 0.2 | 0.0 | [0.0 ; | 0.1] |
| Ø number / year | 283 | 0.0 | ± 0.2 | 0.0 | [0.0 ; | 0.0] | 21,170 | 0.0 | ± 0.0 | 0.0 | [0.0 ; | 0.0] | 0.0 | ± 0.2 | 0.0 | [0.0 ; | 0.0] |
| **SPECT** total | 283 | 0.0 | ± 0.1 | 0.0 | [0.0 ; | 0.0] | 21,170 | 0.0 | ± 0.1 | 0.0 | [0.0 ; | 0.0] | 0.0 | ± 0.1 | 0.0 | [-0.0 ; | 0.0] |
| Ø number / year | 283 | 0.0 | ± 0.0 | 0.0 | [0.0 ; | 0.0] | 21,170 | 0.0 | ± 0.0 | 0.0 | [0.0 ; | 0.0] | 0.0 | ± 0.0 | 0.0 | [0.0 ; | 0.0] |
| **Scintigraphy** total | 283 | 0.2 | ± 0.6 | 0.0 | [0.2 ; | 0.3] | 21,170 | 0.1 | ± 0.3 | 0.0 | [0.1 ; | 0.1] | 0.2 | ± 0.6 | 0.0 | [0.1 ; | 0.2] |
| Ø number / year | 283 | 0.1 | ± 0.3 | 0.0 | [0.1 ; | 0.1] | 21.170 | 0.0 | ± 0.1 | 0.0 | [0.0 ; | 0.0] | 0.1 | ± 0.3 | 0.0 | [0.0 ; | 0.1] |

*Notes: See Appendix B for the respective outpatient GOP or inpatient OPS codes of the different imaging procedures. The overall total numbers (highlighted in grey) refer to the sum of CT, MRI, PET, SPECT and scintigraphy procedures conducted during the observation period and respectively to the average sum per year of observation. The other values refer to the procedure-specific total numbers and the average values per year of observation.*

Table A7: Number of biopsies

|  | **Cohort** | | | | | | | | | | | | | | | | |
| --- | --- | --- | --- | --- | --- | --- | --- | --- | --- | --- | --- | --- | --- | --- | --- | --- | --- |
|  | **TNAMSE patient** | | | | | | **Control cohort** | | | | | | **Difference** | | | | |
|  | **N** | **Mean** | **± SD** | **Median** | **95% CI** | | **N** | **Mean** | **± SD** | **Median** | **95% CI** | | **Mean** | **± SD** | **Median** | **95% CI** | |
| **Total** | | | | | | |  |  |  |  |  |  |  |  |  |  |  |
| Total biopsies | 1,243 | 0.3 | ± 0.9 | 0.0 | [0.2 ; | 0.3] | 92,078 | 0.0 | ± 0.2 | 0.0 | [0.0 ; | 0.0] | 0.2 | ± 0.9 | 0.0 | [0.2 ; | 0.3] |
| Ø number / year | 1,243 | 0.1 | ± 0.5 | 0.0 | [0.1 ; | 0.1] | 92,078 | 0.0 | ± 0.1 | 0.0 | [0.0 ; | 0.0] | 0.1 | ± 0.5 | 0.0 | [0.1 ; | 0.1] |
| **Sex: male** | | | | | | |  |  |  |  |  |  |  |  |  |  |  |
| Total biopsies | 671 | 0.2 | ± 0.9 | 0.0 | [0.2 ; | 0.3] | 49,697 | 0.0 | ± 0.2 | 0.0 | [0.0 ; | 0.0] | 0.2 | ± 0.9 | 0.0 | [0.2 ; | 0.3] |
| Ø number / year | 671 | 0.1 | ± 0.5 | 0.0 | [0.1 ; | 0.1] | 49,697 | 0.0 | ± 0.1 | 0.0 | [0.0 ; | 0.0] | 0.1 | ± 0.5 | 0.0 | [0.1 ; | 0.1] |
| **Sex: female** | | | | | | |  |  |  |  |  |  |  |  |  |  |  |
| Total biopsies | 572 | 0.3 | ± 1.0 | 0.0 | [0.2 ; | 0.4] | 42,381 | 0.0 | ± 0.2 | 0.0 | [0.0 ; | 0.0] | 0.3 | ± 1.0 | 0.0 | [0.2 ; | 0.4] |
| Ø number / year | 572 | 0.1 | ± 0.4 | 0.0 | [0.1 ; | 0.2] | 42,381 | 0.0 | ± 0.1 | 0.0 | [0.0 ; | 0.0] | 0.1 | ± 0.4 | 0.0 | [0.1 ; | 0.1] |
| **Age: < 1 year** | | | | | | |  |  |  |  |  |  |  |  |  |  |  |
| Total biopsies | 138 | 0.1 | ± 0.6 | 0.0 | [0.0 ; | 0.2] | 9,492 | 0.0 | ± 0.1 | 0.0 | [0.0 ; | 0.0] | 0.1 | ± 0.6 | 0.0 | [0.0 ; | 0.2] |
| Ø number / year | 138 | 0.2 | ± 0.8 | 0.0 | [0.0 ; | 0.3] | 9,492 | 0.0 | ± 0.1 | 0.0 | [0.0 ; | 0.0] | 0.2 | ± 0.8 | 0.0 | [0.0 ; | 0.3] |
| **Age: 1 -17 years** | | | | | | |  |  |  |  |  |  |  |  |  |  |  |
| Total biopsies | 822 | 0.2 | ± 0.7 | 0.0 | [0.1 ; | 0.2] | 61,416 | 0.0 | ± 0.2 | 0.0 | [0.0 ; | 0.0] | 0.2 | ± 0.7 | 0.0 | [0.1 ; | 0.2] |
| Ø number / year | 822 | 0.1 | ± 0.5 | 0.0 | [0.1 ; | 0.1] | 61,416 | 0.0 | ± 0.1 | 0.0 | [0.0 ; | 0.0] | 0.1 | ± 0.5 | 0.0 | [0.1 ; | 0.1] |
| **Age: ≥ 18 years** | | | | | | |  |  |  |  |  |  |  |  |  |  |  |
| Total biopsies | 283 | 0.6 | ± 1.4 | 0.0 | [0.4 ; | 0.7] | 21,170 | 0.1 | ± 0.4 | 0.0 | [0.1 ; | 0.1] | 0.5 | ± 1.4 | 0.0 | [0.3 ; | 0.7] |
| Ø number / year | 283 | 0.2 | ± 0.4 | 0.0 | [0.1 ; | 0.2] | 21,170 | 0.0 | ± 0.1 | 0.0 | [0.0 ; | 0.0] | 0.1 | ± 0.4 | 0.0 | [0.1 ; | 0.2] |

*Notes: See Appendix B for the respective outpatient GOP or inpatient OPS codes of biopsies. Means for number of biopsies refer to all biopsies conducted during the observation period. Furthermore, Ø number/year represent the respective average values per year of observation.*

Table A8: Number of genetic tests

|  | **Cohort** | | | | | | | | | | | | | | | | |
| --- | --- | --- | --- | --- | --- | --- | --- | --- | --- | --- | --- | --- | --- | --- | --- | --- | --- |
|  | **TNAMSE patient** | | | | | | **Control cohort** | | | | | | **Difference** | | | | |
|  | **N** | **Mean** | **± SD** | **Median** | **95% CI** | | **N** | **Mean** | **± SD** | **Median** | **95% CI** | | **Mean** | **± SD** | **Median** | **95% CI** | |
| **Total** | | | | | | |  |  |  |  |  |  |  |  |  |  |  |
| GOP 11 total | 1,243 | 14.7 | ± 37.0 | 0.0 | [12.6 ; | 16.7] | 92,078 | 0.3 | ± 5.4 | 0.0 | [0.3 ; | 0.3] | 14.4 | ± 37.0 | 0.0 | [12.3 ; | 16.4] |
| Ø number / year | 1,243 | 5.2 | ± 17.5 | 0.0 | [4.2 ; | 6.2] | 92,078 | 0.1 | ± 1.9 | 0.0 | [0.1 ; | 0.1] | 5.1 | ± 17.5 | 0.0 | [4.1 ; | 6.1] |
| Total panel | 1,243 | 9.1 | ± 26.3 | 0.0 | [7.7 ; | 10.6] | 92,078 | 0.1 | ± 3.3 | 0.0 | [0.1 ; | 0.2] | 9.0 | ± 26.3 | 0.0 | [7.5 ; | 10.5] |
| Ø panel / year | 1,243 | 3.7 | ± 15.5 | 0.0 | [2.8 ; | 4.5] | 92,078 | 0.1 | ± 1.5 | 0.0 | [0.0 ; | 0.1] | 3.6 | ± 15.5 | 0.0 | [2.8 ; | 4.5] |
| **Sex: male** | | | | | | |  |  |  |  |  |  |  |  |  |  |  |
| GOP 11 total | 671 | 16.4 | ± 39.7 | 0.0 | [13.4 ; | 19.4] | 49,697 | 0.3 | ± 6.1 | 0.0 | [0.3 ; | 0.4] | 16.1 | ± 39.7 | 0.0 | [13.1 ; | 19.1] |
| Ø number / year | 671 | 5.7 | ± 19.7 | 0.0 | [4.2 ; | 7.2] | 49,697 | 0.1 | ± 1.9 | 0.0 | [0.1 ; | 0.1] | 5.6 | ± 19.7 | 0.0 | [4.1 ; | 7.1] |
| Total panel | 671 | 9.8 | ± 27.4 | 0.0 | [7.8 ; | 11.9] | 49,697 | 0.2 | ± 3.6 | 0.0 | [0.1 ; | 0.2] | 9.7 | ± 27.4 | 0.0 | [7.6 ; | 11.8] |
| Ø panel / year | 671 | 4.0 | ± 17.6 | 0.0 | [2.7 ; | 5.3] | 49,697 | 0.1 | ± 1.4 | 0.0 | [0.0 ; | 0.1] | 3.9 | ± 17.6 | 0.0 | [2.6 ; | 5.3] |
| **Sex: female** | | | | | | |  |  |  |  |  |  |  |  |  |  |  |
| GOP 11 total | 572 | 12.6 | ± 33.4 | 0.0 | [9.8 ; | 15.3] | 42,381 | 0.3 | ± 4.4 | 0.0 | [0.2 ; | 0.3] | 12.3 | ± 33.4 | 0.0 | [9.6 ; | 15.1] |
| Ø number / year | 572 | 4.5 | ± 14.6 | 0.0 | [3.3 ; | 5.7] | 42,381 | 0.1 | ± 1.9 | 0.0 | [0.1 ; | 0.1] | 4.5 | ± 14.6 | 0.0 | [3.3 ; | 5.7] |
| Total panel | 572 | 8.3 | ± 25.0 | 0.0 | [6.2 ; | 10.3] | 42,381 | 0.1 | ± 3.0 | 0.0 | [0.1 ; | 0.1] | 8.2 | ± 25.0 | 0.0 | [6.1 ; | 10.2] |
| Ø panel / year | 572 | 3.3 | ± 12.6 | 0.0 | [2.3 ; | 4.3] | 42,381 | 0.1 | ± 1.6 | 0.0 | [0.0 ; | 0.1] | 3.3 | ± 12.6 | 0.0 | [2.2 ; | 4.3] |
| **Age: < 1 year** | | | | | | |  |  |  |  |  |  |  |  |  |  |  |
| GOP 11 total | 138 | 5.0 | ± 19.2 | 0.0 | [1.8 ; | 8.2] | 9,492 | 0.1 | ± 2.1 | 0.0 | [0.0 ; | 0.1] | 5.0 | ± 19.2 | 0.0 | [1.7 ; | 8.2] |
| Ø number / year | 138 | 4.5 | ± 17.6 | 0.0 | [1.6 ; | 7.5] | 9,492 | 0.1 | ± 2.0 | 0.0 | [0.0 ; | 0.1] | 4.5 | ± 17.6 | 0.0 | [1.5 ; | 7.4] |
| Total panel | 138 | 4.4 | ± 17.8 | 0.0 | [1.4 ; | 7.4] | 9,492 | 0.1 | ± 1.8 | 0.0 | [0.0 ; | 0.1] | 4.3 | ± 17.8 | 0.0 | [1.3 ; | 7.3] |
| Ø panel / year | 138 | 4.0 | ± 16.3 | 0.0 | [1.2 ; | 6.7] | 9,492 | 0.1 | ± 1.7 | 0.0 | [0.0 ; | 0.1] | 3.9 | ± 16.3 | 0.0 | [1.2 ; | 6.7] |
| **Age: 1 -17 years** | | | | | | |  |  |  |  |  |  |  |  |  |  |  |
| GOP 11... total | 822 | 17.9 | ± 40.1 | 0.0 | [15.1 ; | 20.6] | 61,416 | 0.4 | ± 6.1 | 0.0 | [0.3 ; | 0.4] | 17.5 | ± 40.2 | 0.0 | [14.8 ; | 20.3] |
| Ø number / year | 822 | 6.1 | ± 19.4 | 0.0 | [4.8 ; | 7.5] | 61,416 | 0.1 | ± 2.1 | 0.0 | [0.1 ; | 0.1] | 6.0 | ± 19.5 | 0.0 | [4.7 ; | 7.4] |
| Total panel | 822 | 11.2 | ± 28.7 | 0.0 | [9.2 ; | 13.2] | 61,416 | 0.2 | ± 3.7 | 0.0 | [0.1 ; | 0.2] | 11.0 | ± 28.7 | 0.0 | [9.1 ; | 13.0] |
| Ø panel / year | 822 | 4.3 | ± 17.2 | 0.0 | [3.2 ; | 5.5] | 61,416 | 0.1 | ± 1.6 | 0.0 | [0.1 ; | 0.1] | 4.3 | ± 17.2 | 0.0 | [3.1 ; | 5.4] |
| **Age: ≥ 18 years** | | | | | | |  |  |  |  |  |  |  |  |  |  |  |
| GOP 11 total | 283 | 10.0 | ± 32.5 | 0.0 | [6.2 ; | 13.8] | 21,170 | 0.2 | ± 4.1 | 0.0 | [0.2 ; | 0.3] | 9.8 | ± 32.4 | 0.0 | [6.0 ; | 13.6] |
| Ø number / year | 283 | 2.7 | ± 9.5 | 0.0 | [1.6 ; | 3.8] | 21,170 | 0.1 | ± 1.3 | 0.0 | [0.0 ; | 0.1] | 2.6 | ± 9.5 | 0.0 | [1.5 ; | 3.7] |
| Total panel | 283 | 5.4 | ± 21.6 | 0.0 | [2.9 ; | 7.9] | 21,170 | 0.1 | ± 2.4 | 0.0 | [0.1 ; | 0.1] | 5.3 | ± 21.5 | 0.0 | [2.8 ; | 7.8] |
| Ø panel / year | 283 | 1.6 | ± 7.6 | 0.0 | [0.8 ; | 2.5] | 21,170 | 0.0 | ± 0.8 | 0.0 | [0.0 ; | 0.0] | 1.6 | ± 7.6 | 0.0 | [0.7 ; | 2.5] |

*Notes: The number of genetic tests is reflected by the number of all fee schedule items (GOP) of the German Uniform Assessment Standard (EBM) that begin with the two digits “11”. . Means for number of genetic tests refer to all genetic tests conducted during the observation period. Furthermore, Ø number/year represent the respective average values per year of observation.*

Table A9: Number of laboratory diagnostics with amount per GOP ≥ 25€

|  | **Cohort** | | | | | | | | | | | | | | | | |
| --- | --- | --- | --- | --- | --- | --- | --- | --- | --- | --- | --- | --- | --- | --- | --- | --- | --- |
|  | **TNAMSE patient** | | | | | | **Control cohort** | | | | | | **Difference** | | | | |
|  | **N** | **Mean** | **± SD** | **Median** | **95% CI** | | **N** | **Mean** | **± SD** | **Median** | **95% CI** | | **Mean** | **± SD** | **Median** | **95% CI** | |
| **Total** | | | | | | |  |  |  |  |  |  |  |  |  |  |  |
| Total number | 1,243 | 1.3 | ± 5.6 | 0.0 | [1.0 ; | 1.7] | 92,078 | 0.1 | ± 1.4 | 0.0 | [0.1 ; | 0.1] | 1.2 | ± 5.6 | 0.0 | [0.9 ; | 1.5] |
| Ø number / year | 1,243 | 0.4 | ± 1.3 | 0.0 | [0.3 ; | 0.5] | 92,078 | 0.0 | ± 0.4 | 0.0 | [0.0 ; | 0.0] | 0.3 | ± 1.3 | 0.0 | [0.3 ; | 0.4] |
| **Sex: male** | | | | | | |  |  |  |  |  |  |  |  |  |  |  |
| Total number | 671 | 1.3 | ± 5.9 | 0.0 | [0.8 ; | 1.7] | 49,697 | 0.1 | ± 1.1 | 0.0 | [0.1 ; | 0.1] | 1.2 | ± 5.9 | 0.0 | [0.8 ; | 1.6] |
| Ø number / year | 671 | 0.4 | ± 1.3 | 0.0 | [0.3 ; | 0.5] | 49,697 | 0.0 | ± 0.4 | 0.0 | [0.0 ; | 0.0] | 0.3 | ± 1.3 | 0.0 | [0.2 ; | 0.4] |
| **Sex: female** | | | | | | |  |  |  |  |  |  |  |  |  |  |  |
| Total number | 572 | 1.4 | ± 5.2 | 0.0 | [1.0 ; | 1.8] | 42,381 | 0.2 | ± 1.6 | 0.0 | [0.2 ; | 0.2] | 1.2 | ± 5.1 | 0.0 | [0.8 ; | 1.7] |
| Ø number / year | 572 | 0.4 | ± 1.4 | 0.0 | [0.3 ; | 0.5] | 42,381 | 0.1 | ± 0.4 | 0.0 | [0.0 ; | 0.1] | 0.4 | ± 1.3 | 0.0 | [0.3 ; | 0.5] |
| **Age: < 1 year** | | | | | | |  |  |  |  |  |  |  |  |  |  |  |
| Total number | 138 | 0.2 | ± 0.7 | 0.0 | [0.1 ; | 0.3] | 9,492 | 0.0 | ± 0.2 | 0.0 | [0.0 ; | 0.0] | 0.2 | ± 0.7 | 0.0 | [0.1 ; | 0.3] |
| Ø number / year | 138 | 0.2 | ± 1.1 | 0.0 | [0.0 ; | 0.4] | 9,492 | 0.0 | ± 0.2 | 0.0 | [0.0 ; | 0.0] | 0.2 | ± 1.1 | 0.0 | [0.0 ; | 0.4] |
| **Age: 1 -17 years** | | | | | | |  |  |  |  |  |  |  |  |  |  |  |
| Total number | 822 | 1.0 | ± 4.7 | 0.0 | [0.7 ; | 1.3] | 61,416 | 0.1 | ± 0.7 | 0.0 | [0.1 ; | 0.1] | 0.9 | ± 4.7 | 0.0 | [0.6 ; | 1.2] |
| Ø number / year | 822 | 0.3 | ± 1.1 | 0.0 | [0.2 ; | 0.4] | 61,416 | 0.0 | ± 0.3 | 0.0 | [0.0 ; | 0.0] | 0.3 | ± 1.1 | 0.0 | [0.2 ; | 0.3] |
| **Age: ≥ 18 years** | | | | | | |  |  |  |  |  |  |  |  |  |  |  |
| Total number | 283 | 2.9 | ± 8.4 | 0.0 | [1.9 ; | 3.9] | 21,170 | 0.4 | ± 2.5 | 0.0 | [0.3 ; | 0.4] | 2.6 | ± 8.4 | 0.0 | [1.6 ; | 3.5] |
| Ø number / year | 283 | 0.8 | ± 1.9 | 0.0 | [0.6 ; | 1.0] | 21,170 | 0.1 | ± 0.6 | 0.0 | [0.1 ; | 0.1] | 0.7 | ± 1.9 | 0.0 | [0.5 ; | 0.9] |

*Notes: See Appendix B for the respective outpatient GOP codes of laboratory diagnostics. Means for number of laboratory diagnostics refer to all laboratory diagnostics conducted during the observation period. Furthermore, Ø number/year represent the respective average values per year of observation.*

Table A10: Inpatient costs

|  | **Cohort** | | | | | | | | | | | | | | | | |
| --- | --- | --- | --- | --- | --- | --- | --- | --- | --- | --- | --- | --- | --- | --- | --- | --- | --- |
|  | **TNAMSE patient** | | | | | | **Control cohort** | | | | | | **Difference** | | | | |
|  | **N** | **Mean** | **± SD** | **Median** | **95% CI** | | **N** | **Mean** | **± SD** | **Median** | **95% CI** | | **Mean** | **± SD** | **Median** | **95% CI** | |
| **Total** | | | | | | |  |  |  |  |  |  |  |  |  |  |  |
| Total costs | 1,243 | 16,983 | ± 43,080 | 4,264 | [14,585 ; | 19,380] | 92,078 | 1,809 | ± 11,489 | 0 | [1,735 ; | 1,883] | 15,174 | ± 42,919 | 2,737 | [12,786 ; | 17,562] |
| Ø costs / year observed | 1,243 | 10,527 | ± 38,700 | 1,569 | [8,374 ; | 12,681] | 92,078 | 772 | ± 6,942 | 0 | [728 ; | 817] | 9,726 | ± 38,378 | 962 | [7,590 ; | 11,861] |
| Ø costs / years insured | 1,243 | 16,797 | ± 74,117 | 1,640 | [12,673 ; | 20,922] | 92,078 | 1,094 | ± 14,281 | 0 | [1,001 ; | 1,186] | 15,648 | ± 73,337 | 1,016 | [11,567 ; | 19,729] |
| **Sex: male** | | | | | | |  |  |  |  |  |  |  |  |  |  |  |
| Total costs | 671 | 17,986 | ± 43,225 | 4,071 | [14,710 ; | 21,262] | 49,697 | 1,893 | ± 12,539 | 0 | [1,782 ; | 2,003] | 16,094 | ± 42,982 | 2,549 | [12,836 ; | 19,352] |
| Ø costs / year observed | 671 | 10,439 | ± 38,546 | 1,438 | [7,517 ; | 13,360] | 49,697 | 821 | ± 7,592 | 0 | [755 ; | 888] | 9,584 | ± 38,130 | 885 | [6,694 ; | 12,474] |
| Ø costs / years insured | 671 | 16,648 | ± 65,763 | 1,492 | [11,663 ; | 21,633] | 49,697 | 1,226 | ± 17,142 | 0 | [1,075 ; | 1,377] | 15,359 | ± 64,698 | 923 | [10,454 ; | 20,263] |
| **Sex: female** | | | | | | |  |  |  |  |  |  |  |  |  |  |  |
| Total costs | 572 | 15,806 | ± 42,918 | 4,734 | [12,281 ; | 19,330] | 42,381 | 1,711 | ± 10,119 | 0 | [1,615 ; | 1,808] | 14,095 | ± 42,858 | 3,114 | [10,576 ; | 17,615] |
| Ø costs / year observed | 572 | 10,631 | ± 38,913 | 1,708 | [7,435 ; | 13,827] | 42,381 | 715 | ± 6,092 | 0 | [657 ; | 773] | 9,892 | ± 38,700 | 1,047 | [6,714 ; | 13,070] |
| Ø costs / years insured | 572 | 16,973 | ± 82,909 | 1,762 | [10,164 ; | 23,781] | 42,381 | 939 | ± 9,922 | 0 | [844 ; | 1,033] | 15,988 | ± 82,382 | 1,115 | [9,222 ; | 22,753] |
| **Age: < 1 year** | | | | | | |  |  |  |  |  |  |  |  |  |  |  |
| Total costs | 138 | 31,595 | ± 54,884 | 12,939 | [22,356 ; | 40,834] | 9,492 | 2,114 | ± 13,655 | 0 | [1,840 ; | 2,389] | 29,512 | ± 54,927 | 11,351 | [20,266 ; | 38,758] |
| Ø costs / year observed | 138 | 50,025 | ± 99,680 | 19,883 | [33,246 ; | 66,804] | 9,492 | 2,590 | ± 17,776 | 0 | [2,233 ; | 2,948] | 47,319 | ± 99,426 | 16,585 | [30,582 ; | 64,055] |
| Ø costs / years insured | 138 | 103,999 | ± 196,078 | 28,255 | [70,993 ; | 137,005] | 9,492 | 5,545 | ± 42,238 | 0 | [4,696 ; | 6,395] | 98,308 | ± 195,693 | 23,667 | [65,367 ; | 131,249] |
| **Age: 1 -17 years** | | | | | | |  |  |  |  |  |  |  |  |  |  |  |
| Total costs | 822 | 15,751 | ± 44,265 | 3,092 | [12,721 ; | 18,782] | 61,416 | 1,532 | ± 11,720 | 0 | [1,439 ; | 1,625] | 14,219 | ± 44,020 | 2,217 | [11,205 ; | 17,233] |
| Ø costs / year observed | 822 | 6,253 | ± 17,137 | 1,115 | [5,080 ; | 7,426] | 61,416 | 511 | ± 4,407 | 0 | [476 ; | 546] | 5,742 | ± 17,083 | 669 | [4,572 ; | 6,911] |
| Ø costs / years insured | 822 | 6,722 | ± 20,933 | 1,176 | [5,288 ; | 8,155] | 61,416 | 548 | ± 4,899 | 0 | [509 ; | 587] | 6,173 | ± 20,856 | 694 | [4,746 ; | 7,601] |
| **Age: ≥ 18 years** | | | | | | |  |  |  |  |  |  |  |  |  |  |  |
| Total costs | 283 | 13,434 | ± 29,737 | 5,360 | [9,954 ; | 16,914] | 21,170 | 2,477 | ± 9,555 | 0 | [2,348 ; | 2,605] | 10,957 | ± 29,743 | 3,090 | [7,477 ; | 14,438] |
| Ø costs / year observed | 283 | 3,682 | ± 7,096 | 1,659 | [2,851 ; | 4,512] | 21,170 | 716 | ± 3,150 | 0 | [674 ; | 759] | 2,966 | ± 7,141 | 904 | [2,130 ; | 3,801] |
| Ø costs / years insured | 283 | 3,541 | ± 6,836 | 1,678 | [2,742 ; | 4,341] | 21,170 | 681 | ± 2,757 | 0 | [644 ; | 718] | 2,860 | ± 6,871 | 950 | [2,057 ; | 3,664] |

*Notes: Total inpatient costs refer to the sum of all specific costs incurred during the observation period. Ø costs /year observed represent the respective average costs per year of observation.
Ø costs /year insured takes into account the actual number of days an individual was insured in the respective observation year. Costs in Euro.*

Table A11: Outpatient costs

|  | **Cohort** | | | | | | | | | | | | | | | | |
| --- | --- | --- | --- | --- | --- | --- | --- | --- | --- | --- | --- | --- | --- | --- | --- | --- | --- |
|  | **TNAMSE patient** | | | | | | **Control cohort** | | | | | | **Difference** | | | | |
|  | **N** | **Mean** | **± SD** | **Median** | **95% CI** | | **N** | **Mean** | **± SD** | **Median** | **95% CI** | | **Mean** | **± SD** | **Median** | **95% CI** | |
| **Total** | | | | | | |  |  |  |  |  |  |  |  |  |  |  |
| Total costs | 1,243 | 3,197 | ± 8,607 | 1,575 | [2,718 ; | 3,676] | 92,078 | 1,010 | ± 1,611 | 593 | [1,000 ; | 1,021] | 2,194 | ± 8,397 | 528 | [1,726 ; | 2,661] |
| Ø costs / year observed | 1,243 | 959 | ± 1,888 | 555 | [854 ; | 1,064] | 92,078 | 316 | ± 433 | 242 | [313 ; | 319] | 644 | ± 1,851 | 207 | [541 ; | 747] |
| Ø costs / years insured | 1,243 | 1,068 | ± 3,327 | 587 | [883 ; | 1,253] | 92,078 | 324 | ± 426 | 245 | [322 ; | 327] | 743 | ± 3,305 | 231 | [559 ; | 927] |
| **Sex: male** | | | | | | |  |  |  |  |  |  |  |  |  |  |  |
| Total costs | 671 | 3,292 | ± 10,753 | 1,611 | [2,477 ; | 4,107] | 49,697 | 953 | ± 1,308 | 591 | [941 ; | 964] | 2,346 | ± 10,613 | 540 | [1,542 ; | 3,150] |
| Ø costs / year observed | 671 | 916 | ± 2,030 | 523 | [762 ; | 1,069] | 49,697 | 293 | ± 367 | 234 | [289 ; | 296] | 623 | ± 2,012 | 191 | [471 ; | 776] |
| Ø costs / years insured | 671 | 991 | ± 2,445 | 555 | [806 ; | 1,176] | 49,697 | 304 | ± 370 | 236 | [300 ; | 307] | 686 | ± 2,422 | 221 | [503 ; | 870] |
| **Sex: female** | | | | | | |  |  |  |  |  |  |  |  |  |  |  |
| Total costs | 572 | 3,084 | ± 5,044 | 1,515 | [2,670 ; | 3,498] | 42,381 | 1,078 | ± 1,904 | 596 | [1,060 ; | 1,096] | 2,015 | ± 4,597 | 500 | [1,637 ; | 2,393] |
| Ø costs / year observed | 572 | 1,010 | ± 1,707 | 589 | [870 ; | 1,151] | 42,381 | 343 | ± 499 | 252 | [338 ; | 347] | 669 | ± 1,645 | 222 | [534 ; | 804] |
| Ø costs / years insured | 572 | 1,158 | ± 4,128 | 620 | [819 ; | 1,497] | 42,381 | 348 | ± 483 | 256 | [344 ; | 353] | 810 | ± 4,108 | 239 | [473 ; | 1,147] |
| **Age: < 1 year** | | | | | | |  |  |  |  |  |  |  |  |  |  |  |
| Total costs | 138 | 509 | ± 1,005 | 151 | [340 ; | 678] | 9,492 | 252 | ± 267 | 189 | [247 ; | 258] | 268 | ± 943 | 5 | [110 ; | 427] |
| Ø costs / year observed | 138 | 563 | ± 1,056 | 210 | [386 ; | 741] | 9,492 | 291 | ± 281 | 259 | [286 ; | 297] | 278 | ± 1,026 | 10 | [105 ; | 451] |
| Ø costs / years insured | 138 | 1,045 | ± 2,819 | 468 | [570 ; | 1,520] | 9,492 | 415 | ± 409 | 422 | [407 ; | 423] | 636 | ± 2,773 | 39 | [169 ; | 1,103] |
| **Age: 1 -17 years** | | | | | | |  |  |  |  |  |  |  |  |  |  |  |
| Total costs | 822 | 2,874 | ± 8,261 | 1,668 | [2,308 ; | 3,439] | 61,416 | 949 | ± 1,181 | 677 | [940 ; | 959] | 1,923 | ± 8,119 | 552 | [1,368 ; | 2,479] |
| Ø costs / year observed | 822 | 853 | ± 1,824 | 529 | [728 ; | 978] | 61,416 | 280 | ± 301 | 239 | [278 ; | 283] | 572 | ± 1,804 | 199 | [448 ; | 695] |
| Ø costs / years insured | 822 | 951 | ± 3,683 | 536 | [699 ; | 1,203] | 61,416 | 281 | ± 301 | 235 | [279 ; | 284] | 669 | ± 3,673 | 220 | [418 ; | 921] |
| **Age: ≥ 18 years** | | | | | | |  |  |  |  |  |  |  |  |  |  |  |
| Total costs | 283 | 5,445 | ± 10,872 | 3,242 | [4,173 ; | 6,717] | 21,170 | 1,527 | ± 2,584 | 815 | [1,492 ; | 1,562] | 3,918 | ± 10,638 | 1,419 | [2,673 ; | 5,162] |
| Ø costs / year observed | 283 | 1,462 | ± 2,262 | 986 | [1,197 ; | 1,726] | 21,170 | 429 | ± 708 | 253 | [419 ; | 439] | 1,033 | ± 2,207 | 516 | [775 ; | 1,291] |
| Ø costs / years insured | 283 | 1,417 | ± 2,286 | 978 | [1,150 ; | 1,685] | 21,170 | 408 | ± 662 | 241 | [399 ; | 417] | 1,009 | ± 2,238 | 512 | [747 ; | 1,271] |

*Notes: Total outpatient costs refer to the sum of all specific costs incurred during the observation period. Ø costs /year observed represent the respective average costs per year of observation.
Ø costs /year insured takes into account the actual number of days an individual was insured in the respective observation year. Costs in Euro.*

Table A12: Costs for prescription drugs filled at pharmacies

|  | **Cohort** | | | | | | | | | | | | | | | | |
| --- | --- | --- | --- | --- | --- | --- | --- | --- | --- | --- | --- | --- | --- | --- | --- | --- | --- |
|  | **TNAMSE patient** | | | | | | **Control cohort** | | | | | | **Difference** | | | | |
|  | **N** | **Mean** | **± SD** | **Median** | **95% CI** | | **N** | **Mean** | **± SD** | **Median** | **95% CI** | | **Mean** | **± SD** | **Median** | **95% CI** | |
| **Total** | | | | | | |  |  |  |  |  |  |  |  |  |  |  |
| Total costs | 1,243 | 6,820 | ± 29,630 | 435 | [5,171 ; | 8,468] | 92,078 | 741 | ± 10,311 | 134 | [675 ; | 808] | 6,085 | ± 29,602 | 31 | [4,438 ; | 7,732] |
| Ø costs / year observed | 1,243 | 1,661 | ± 6,220 | 159 | [1,315 ; | 2,007] | 92,078 | 223 | ± 2,834 | 49 | [205 ; | 242] | 1,440 | ± 6,233 | 14 | [1,093 ; | 1,786] |
| Ø costs / years insured | 1,243 | 1,708 | ± 6,401 | 159 | [1,352 ; | 2,064] | 92,078 | 221 | ± 2,782 | 51 | [203 ; | 239] | 1,488 | ± 6,415 | 15 | [1,131 ; | 1,845] |
| **Sex: male** | | | | | | |  |  |  |  |  |  |  |  |  |  |  |
| Total costs | 671 | 7,252 | ± 27,402 | 427 | [5,175 ; | 9,329] | 49,697 | 710 | ± 13,098 | 129 | [595 ; | 825] | 6,548 | ± 27,399 | 34 | [4,471 ; | 8,625] |
| Ø costs / year observed | 671 | 1,688 | ± 5,910 | 149 | [1,240 ; | 2,136] | 49,697 | 214 | ± 3,594 | 46 | [182 ; | 245] | 1,476 | ± 5,934 | 16 | [1,026 ; | 1,926] |
| Ø costs / years insured | 671 | 1,741 | ± 5,805 | 153 | [1,301 ; | 2,181] | 49,697 | 213 | ± 3,539 | 48 | [182 ; | 244] | 1,530 | ± 5,829 | 22 | [1,088 ; | 1,972] |
| **Sex: female** | | | | | | |  |  |  |  |  |  |  |  |  |  |  |
| Total costs | 572 | 6,313 | ± 32,065 | 459 | [3,679 ; | 8,946] | 42,381 | 778 | ± 5,463 | 141 | [726 ; | 830] | 5,542 | ± 32,010 | 29 | [2,913 ; | 8,171] |
| Ø costs / year observed | 572 | 1,629 | ± 6,570 | 165 | [1,090 ; | 2,169] | 42,381 | 234 | ± 1,517 | 53 | [220 ; | 249] | 1,397 | ± 6,572 | 12 | [857 ; | 1,937] |
| Ø costs / years insured | 572 | 1,669 | ± 7,042 | 166 | [1,090 ; | 2,247] | 42,381 | 231 | ± 1,457 | 55 | [217 ; | 245] | 1,440 | ± 7,046 | 12 | [861 ; | 2,018] |
| **Age: < 1 year** | | | | | | |  |  |  |  |  |  |  |  |  |  |  |
| Total costs | 138 | 494 | ± 1,158 | 47 | [299 ; | 688] | 9,492 | 93 | ± 501 | 25 | [83 ; | 103] | 405 | ± 1,147 | -9 | [212 ; | 598] |
| Ø costs / year observed | 138 | 636 | ± 1,882 | 59 | [319 ; | 953] | 9,492 | 100 | ± 508 | 30 | [90 ; | 110] | 539 | ± 1,854 | -17 | [227 ; | 851] |
| Ø costs / years insured | 138 | 789 | ± 1,732 | 97 | [497 ; | 1,080] | 9,492 | 143 | ± 767 | 45 | [128 ; | 158] | 652 | ± 1,718 | -14 | [362 ; | 941] |
| **Age: 1 -17 years** | | | | | | |  |  |  |  |  |  |  |  |  |  |  |
| Total costs | 822 | 7,257 | ± 32,177 | 409 | [5,054 ; | 9,460] | 61,416 | 507 | ± 11,349 | 148 | [417 ; | 596] | 6,751 | ± 32,143 | 60 | [4,550 ; | 8,951] |
| Ø costs / year observed | 822 | 1,738 | ± 6,891 | 140 | [1,266 ; | 2,210] | 61,416 | 154 | ± 3,147 | 50 | [129 ; | 179] | 1,585 | ± 6,900 | 24 | [1,112 ; | 2,057] |
| Ø costs / years insured | 822 | 1,795 | ± 7,174 | 141 | [1,304 ; | 2,287] | 61,416 | 152 | ± 3,106 | 50 | [127 ; | 176] | 1,644 | ± 7,183 | 33 | [1,152 ; | 2,136] |
| **Age: ≥ 18 years** | | | | | | |  |  |  |  |  |  |  |  |  |  |  |
| Total costs | 283 | 8,634 | ± 28,782 | 1,101 | [5,266 ; | 12,002] | 21,170 | 1,713 | ± 9,349 | 219 | [1,588 ; | 1,839] | 6,922 | ± 28,862 | -44 | [3,545 ; | 10,299] |
| Ø costs / year observed | 283 | 1,938 | ± 5,462 | 358 | [1,299 ; | 2,577] | 21,170 | 480 | ± 2,449 | 63 | [447 ; | 513] | 1,458 | ± 5,513 | -14 | [813 ; | 2,103] |
| Ø costs / years insured | 283 | 1,902 | ± 5,359 | 330 | [1,275 ; | 2,529] | 21,170 | 458 | ± 2,307 | 61 | [427 ; | 489] | 1,444 | ± 5,405 | -8 | [812 ; | 2,077] |

*Notes: Total costs of prescription drugs filled at pharmacies refer to the sum of all specific costs incurred during the observation period. Ø costs /year observed represent the respective average costs per year of observation. Ø costs /year insured takes into account the actual number of days an individual was insured in the respective observation year. Costs in Euro.*

Table A13: Total costs

|  | **Cohort** | | | | | | | | | | | | | | | | |
| --- | --- | --- | --- | --- | --- | --- | --- | --- | --- | --- | --- | --- | --- | --- | --- | --- | --- |
|  | **TNAMSE patient** | | | | | | **Control cohort** | | | | | | **Difference** | | | | |
|  | **N** | **Mean** | **± SD** | **Median** | **95% CI** | | **N** | **Mean** | **± SD** | **Median** | **95% CI** | | **Mean** | **± SD** | **Median** | **95% CI** | |
| **Total** | | | | | | |  |  |  |  |  |  |  |  |  |  |  |
| Total costs | 1,243 | 26,999 | ± 58,362 | 8,961 | [23,751 ; | 30,247] | 92,078 | 3,561 | ± 16,381 | 1092 | [3,455 ; | 3,667] | 23,453 | ± 58,075 | 5,426 | [20,221 ; | 26,685] |
| Ø costs / year observed | 1,243 | 13,147 | ± 39,339 | 3,150 | [10,958 ; | 15,337] | 92,078 | 1,311 | ± 7,679 | 384 | [1,262 ; | 1,361] | 11,810 | ± 39,067 | 2,033 | [9,636 ; | 13,983] |
| Ø costs / years insured | 1,243 | 19,573 | ± 74,458 | 3,197 | [15,430 ; | 23,716] | 92,078 | 1,639 | ± 14,657 | 394 | [1,544 ; | 1,734] | 17,879 | ± 73,690 | 2,122 | [13,779 ; | 21,980] |
| **Sex: male** | | | | | | |  |  |  |  |  |  |  |  |  |  |  |
| Total costs | 671 | 28,530 | ± 59,599 | 8,886 | [24,013 ; | 33,048] | 49,697 | 3,555 | ± 18,977 | 1110 | [3,388 ; | 3,722] | 24,988 | ± 59,325 | 5,389 | [20,491 ; | 29,485] |
| Ø costs / year observed | 671 | 13,042 | ± 39,181 | 3,006 | [10,073 ; | 16,012] | 49,697 | 1,328 | ± 8,570 | 377 | [1,253 ; | 1,403] | 11,683 | ± 38,818 | 1,875 | [8,741 ; | 14,625] |
| Ø costs / years insured | 671 | 19,380 | ± 66,112 | 2,996 | [14,369 ; | 24,391] | 49,697 | 1,742 | ± 17,595 | 385 | [1,588 ; | 1,897] | 17,575 | ± 65,059 | 1,851 | [12,643 ; | 22,506] |
| **Sex: female** | | | | | | |  |  |  |  |  |  |  |  |  |  |  |
| Total costs | 572 | 25,202 | ± 56,877 | 9,010 | [20,532 ; | 29,873] | 42,381 | 3,567 | ± 12,676 | 1072 | [3,447 ; | 3,688] | 21,652 | ± 56,573 | 5,454 | [17,006 ; | 26,298] |
| Ø costs / year observed | 572 | 13,271 | ± 39,558 | 3,394 | [10,022 ; | 16,519] | 42,381 | 1,292 | ± 6,480 | 393 | [1,230 ; | 1,354] | 11,958 | ± 39,391 | 2,236 | [8,723 ; | 15,193] |
| Ø costs / years insured | 572 | 19,799 | ± 83,248 | 3,737 | [12,962 ; | 26,636] | 42,381 | 1,518 | ± 10,184 | 405 | [1,421 ; | 1,615] | 18,237 | ± 82,732 | 2,453 | [11,443 ; | 25,031] |
| **Age: < 1 year** | | | | | | |  |  |  |  |  |  |  |  |  |  |  |
| Total costs | 138 | 32,598 | ± 54,940 | 13,918 | [23,350 ; | 41,846] | 9,492 | 2,460 | ± 13,805 | 371 | [2,182 ; | 2,738] | 30,186 | ± 54,981 | 12,073 | [20,931 ; | 39,440] |
| Ø costs / year observed | 138 | 51,224 | ± 99,691 | 20,366 | [34,443 ; | 68,005] | 9,492 | 2,982 | ± 17,885 | 454 | [2,622 ; | 3,342] | 48,136 | ± 99,460 | 16,955 | [31,394 ; | 64,878] |
| Ø costs / years insured | 138 | 105,832 | ± 195,801 | 31,129 | [72,873 ; | 138,791] | 9,492 | 6,104 | ± 42,326 | 608 | [5,252 ; | 6,955] | 99,596 | ± 195,402 | 26,311 | [66,704 ; | 132,488] |
| **Age: 1 -17 years** | | | | | | |  |  |  |  |  |  |  |  |  |  |  |
| Total costs | 822 | 25,882 | ± 62,116 | 6,984 | [21,629 ; | 30,135] | 61,416 | 2,988 | ± 17,033 | 1083 | [2,853 ; | 3,123] | 22,893 | ± 61,699 | 4,196 | [18,669 ; | 27,117] |
| Ø costs / year observed | 822 | 8,844 | ± 19,324 | 2,544 | [7,521 ; | 10,167] | 61,416 | 945 | ± 5,612 | 357 | [901 ; | 989] | 7,898 | ± 19,282 | 1,670 | [6,578 ; | 9,218] |
| Ø costs / years insured | 822 | 9,468 | ± 23,409 | 2,500 | [7,865 ; | 11,071] | 61,416 | 981 | ± 6,002 | 354 | [933 ; | 1,028] | 8,487 | ± 23,336 | 1,696 | [6,889 ; | 10,084] |
| **Age: ≥ 18 years** | | | | | | |  |  |  |  |  |  |  |  |  |  |  |
| Total costs | 283 | 27,513 | ± 47,778 | 12,995 | [21,922 ; | 33,103] | 21,170 | 5,717 | ± 15,293 | 1780 | [5,511 ; | 5,923] | 21,797 | ± 47,719 | 6,766 | [16,213 ; | 27,380] |
| Ø costs / year observed | 283 | 7,081 | ± 10,016 | 3,952 | [5,909 ; | 8,253] | 21,170 | 1,625 | ± 4,467 | 517 | [1,565 ; | 1,685] | 5,456 | ± 10,139 | 2,090 | [4,270 ; | 6,643] |
| Ø costs / years insured | 283 | 6,861 | ± 9,782 | 3,752 | [5,716 ; | 8,005] | 21,170 | 1,547 | ± 4,064 | 493 | [1,492 ; | 1,602] | 5,314 | ± 9,889 | 2,129 | [4,157 ; | 6,471] |

*Notes: Here, the overall (total) costs refer to the sum of outpatient, inpatient and prescription drug costs. The total values refer to the sum of all specific costs incurred during the observation period. Ø costs /year observed represent the respective average costs per year of observation. Ø costs /year insured takes into account the actual number of days an individual was insured in the respective observation year. Costs in Euro.*

Table A14: Diagnostic pathway for individuals diagnosed with a rare disease

|  | **Cohort** | | | | | | | | | | | | | | | | |
| --- | --- | --- | --- | --- | --- | --- | --- | --- | --- | --- | --- | --- | --- | --- | --- | --- | --- |
|  | **TNAMSE patient** | | | | | | **Control cohort** | | | | | | **Difference** | | | | |
|  | **N** | **Mean** | **± SD** | **Median** | **95% CI** | | **N** | **Mean** | **± SD** | **Median** | **95% CI** | | **Mean** | **± SD** | **Median** | **95% CI** | |
| **Hospitalization** | | | | | | |  |  |  |  |  |  |  |  |  |  |  |
| Total hospitalization | 313 | 2.6 | ± 3.3 | 1.0 | [2.2 ; | 3.0] | 23.080 | 0.5 | ± 1.5 | 0.0 | [0.5 ; | 0.5] | 2.1 | ± 3.3 | 0.9 | [1.8 ; | 2.5] |
| Ø hospitalizations / year | 313 | 1.6 | ± 5.5 | 0.6 | [1.0 ; | 2.2] | 23.080 | 0.2 | ± 0.6 | 0.0 | [0.2 ; | 0.2] | 1.4 | ± 5.4 | 0.5 | [0.8 ; | 2.0] |
| Total duration [days] | 313 | 17.3 | ± 34.3 | 5.0 | [13.4 ; | 21.1] | 23.080 | 2.4 | ± 12.5 | 0.0 | [2.2 ; | 2.5] | 14.9 | ± 34.3 | 3.0 | [11.1 ; | 18.7] |
| Ø duration / year | 313 | 10.4 | ± 23.0 | 2.2 | [7.8 ; | 12.9] | 23.080 | 1.0 | ± 6.6 | 0.0 | [1.0 ; | 1.1] | 9.3 | ± 22.7 | 1.3 | [6.7 ; | 11.8] |
| **Diagnoses** | | | | | | |  |  |  |  |  |  |  |  |  |  |  |
| Total number | 313 | 42.9 | ± 27.5 | 36.0 | [39.8 ; | 45.9] | 23.080 | 24.9 | ± 18.5 | 21.0 | [24.6 ; | 25.1] | 18.2 | ± 22.4 | 13.7 | [15.7 ; | 20.7] |
| **Outpatient treatment: Number of specialist groups and operating facilities** | | | | | | |  |  |  |  |  |  |  |  |  |  |  |
| Number of specialists | 313 | 6.2 | ± 3.8 | 6.0 | [5.7 ; | 6.6] | 23.080 | 4.0 | ± 3.0 | 3.0 | [4.0 ; | 4.0] | 2.2 | ± 2.8 | 2.0 | [1.9 ; | 2.5] |
| Ø number / year | 313 | 2.8 | ± 2.7 | 2.0 | [2.5 ; | 3.1] | 23.080 | 1.7 | ± 2.4 | 1.3 | [1.7 ; | 1.8] | 1.1 | ± 2.1 | 0.7 | [0.8 ; | 1.3] |
| Number of operating sites | 313 | 9.8 | ± 6.6 | 9.0 | [9.0 ; | 10.5] | 23.080 | 6.0 | ± 4.8 | 5.0 | [5.9 ; | 6.1] | 3.8 | ± 5.0 | 3.3 | [3.3 ; | 4.4] |
| Ø number / year | 313 | 5.2 | ± 15.7 | 3.1 | [3.5 ; | 7.0] | 23.080 | 3.0 | ± 10.7 | 2.0 | [2.9 ; | 3.1] | 2.2 | ± 7.3 | 1.1 | [1.4 ; | 3.0] |
| **Active substances prescribed** | | | | | | |  |  |  |  |  |  |  |  |  |  |  |
| Total number | 313 | 11.2 | ± 8.7 | 10.0 | [10.2 ; | 12.2] | 23.080 | 8.2 | ± 6.9 | 7.0 | [8.1 ; | 8.3] | 3.1 | ± 7.4 | 1.5 | [2.3 ; | 4.0] |
| Ø number / year | 313 | 4.5 | ± 4.4 | 3.6 | [4.0 ; | 5.0] | 23.080 | 3.3 | ± 3.7 | 2.5 | [3.2 ; | 3.3] | 1.2 | ± 4.1 | 0.7 | [0.8 ; | 1.7] |
| **Number of imaging procedures** | | | | | | |  |  |  |  |  |  |  |  |  |  |  |
| **Total** number | 313 | 1.5 | ± 2.5 | 0.0 | [1.2 ; | 1.7] | 23.080 | 0.2 | ± 1.1 | 0.0 | [0.2 ; | 0.2] | 1.3 | ± 2.4 | 0.0 | [1.0 ; | 1.5] |
| Ø number / year | 313 | 0.8 | ± 2.1 | 0.0 | [0.6 ; | 1.0] | 23.080 | 0.1 | ± 0.4 | 0.0 | [0.1 ; | 0.1] | 0.7 | ± 2.0 | 0.0 | [0.5 ; | 1.0] |
| **CT** total | 313 | 0.3 | ± 1.2 | 0.0 | [0.2 ; | 0.4] | 23.080 | 0.1 | ± 0.6 | 0.0 | [0.1 ; | 0.1] | 0.2 | ± 1.1 | 0.0 | [0.1 ; | 0.4] |
| Ø number / year | 313 | 0.1 | ± 0.4 | 0.0 | [0.1 ; | 0.1] | 23.080 | 0.0 | ± 0.2 | 0.0 | [0.0 ; | 0.0] | 0.1 | ± 0.4 | 0.0 | [0.0 ; | 0.1] |
| **MRI** total | 313 | 1.1 | ± 2.0 | 0.0 | [0.9 ; | 1.3] | 23.080 | 0.1 | ± 0.8 | 0.0 | [0.1 ; | 0.2] | 1.0 | ± 1.9 | 0.0 | [0.8 ; | 1.2] |
| Ø number / year | 313 | 0.7 | ± 1.9 | 0.0 | [0.5 ; | 0.9] | 23.080 | 0.0 | ± 0.3 | 0.0 | [0.0 ; | 0.0] | 0.6 | ± 1.9 | 0.0 | [0.4 ; | 0.9] |
| **PET** total | 313 | 0.0 | ± 0.1 | 0.0 | [0.0 ; | 0.0] | 23.080 | 0.0 | ± 0.0 | 0.0 | [0.0 ; | 0.0] | 0.0 | ± 0.1 | 0.0 | [0.0 ; | 0.0] |
| Ø number / year | 313 | 0.0 | ± 0.1 | 0.0 | [0.0 ; | 0.0] | 23.080 | 0.0 | ± 0.0 | 0.0 | [0.0 ; | 0.0] | 0.0 | ± 0.1 | 0.0 | [0.0 ; | 0.0] |
| **SPECT** total | 313 | 0.0 | ± 0.1 | 0.0 | [0.0 ; | 0.0] | 23.080 | 0.0 | ± 0.0 | 0.0 | [0.0 ; | 0.0] | 0.0 | ± 0.1 | 0.0 | [0.0 ; | 0.0] |
| Ø number / year | 313 | 0.0 | ± 0.0 | 0.0 | [0.0 ; | 0.0] | 23.080 | 0.0 | ± 0.0 | 0.0 | [0.0 ; | 0.0] | 0.0 | ± 0.0 | 0.0 | [0.0 ; | 0.0] |
| **Scintigraphy** total | 313 | 0.0 | ± 0.2 | 0.0 | [0.0 ; | 0.0] | 23.080 | 0.0 | ± 0.1 | 0.0 | [0.0 ; | 0.0] | 0.0 | ± 0.2 | 0.0 | [0.0 ; | 0.0] |
| Ø number / year | 313 | 0.0 | ± 0.1 | 0.0 | [0.0 ; | 0.0] | 23.080 | 0.0 | ± 0.1 | 0.0 | [0.0 ; | 0.0] | 0.0 | ± 0.1 | 0.0 | [0.0 ; | 0.0] |

**For a continuation of Table A14, see next page.**

|  | **Cohort** | | | | | | | | | | | | | | | | |
| --- | --- | --- | --- | --- | --- | --- | --- | --- | --- | --- | --- | --- | --- | --- | --- | --- | --- |
|  | **TNAMSE patient** | | | | | | **Control cohort** | | | | | | **Difference** | | | | |
|  | **N** | **Mean** | **± SD** | **Median** | **95% CI** | | **N** | **Mean** | **± SD** | **Median** | **95% CI** | | **Mean** | **± SD** | **Median** | **95% CI** | |
| **Biopsies** | | | | | | |  |  |  |  |  |  |  |  |  |  |  |
| Total biopsies | 313 | 0.2 | ± 0.7 | 0.0 | [0.1 ; | 0.2] | 23.080 | 0.0 | ± 0.2 | 0.0 | [0.0 ; | 0.0] | 0.2 | ± 0.7 | 0.0 | [0.1 ; | 0.2] |
| Ø number / year | 313 | 0.1 | ± 0.5 | 0.0 | [0.0 ; | 0.2] | 23.080 | 0.0 | ± 0.1 | 0.0 | [0.0 ; | 0.0] | 0.1 | ± 0.5 | 0.0 | [0.0 ; | 0.2] |
| **Number of genetic tests** | | | | | | |  |  |  |  |  |  |  |  |  |  |  |
| GOP 11 total | 313 | 12.6 | ± 33.7 | 0.0 | [8.9 ; | 16.4] | 23.080 | 0.3 | ± 4.6 | 0.0 | [0.2 ; | 0.3] | 12.4 | ± 33.7 | 0.0 | [8.6 ; | 16.1] |
| Ø number / year | 313 | 4.3 | ± 13.1 | 0.0 | [2.9 ; | 5.8] | 23.080 | 0.1 | ± 2.1 | 0.0 | [0.1 ; | 0.1] | 4.2 | ± 13.1 | 0.0 | [2.8 ; | 5.7] |
| Total panel | 313 | 7.4 | ± 22.9 | 0.0 | [4.8 ; | 9.9] | 23.080 | 0.1 | ± 3.3 | 0.0 | [0.1 ; | 0.2] | 7.2 | ± 23.0 | 0.0 | [4.7 ; | 9.8] |
| Ø panel / year | 313 | 2.9 | ± 11.0 | 0.0 | [1.7 ; | 4.1] | 23.080 | 0.1 | ± 1.8 | 0.0 | [0.0 ; | 0.1] | 2.9 | ± 11.0 | 0.0 | [1.6 ; | 4.1] |
| **Number of laboratory diagnostics with amount per GOP ≥ 25€** | | | | | | |  |  |  |  |  |  |  |  |  |  |  |
| Total number | 313 | 0.8 | ± 2.2 | 0.0 | [0.5 ; | 1.0] | 23.080 | 0.1 | ± 1.1 | 0.0 | [0.1 ; | 0.1] | 0.7 | ± 2.1 | 0.0 | [0.4 ; | 0.9] |
| Ø number / year | 313 | 0.3 | ± 0.9 | 0.0 | [0.2 ; | 0.4] | 23.080 | 0.0 | ± 0.4 | 0.0 | [0.0 ; | 0.0] | 0.2 | ± 0.9 | 0.0 | [0.1 ; | 0.3] |

*Notes: Here, we show the findings regarding the diagnostic pathway only for individuals, which were diagnosed with a rare disease within the TNAMSE project. For further explanations regarding the different indicators, please see the table note for each specific indicators.*

Table A15: Total costs differentiated by diagnosis type

|  | **Cohort** | | | | | | | | | | | | | | | | | | | | | |
| --- | --- | --- | --- | --- | --- | --- | --- | --- | --- | --- | --- | --- | --- | --- | --- | --- | --- | --- | --- | --- | --- | --- |
|  | **TNAMSE patient** | | | | | | | **Control cohort** | | | | | | | | | **Difference** | | | | | |
|  | **N** | | **Mean** | **± SD** | **Median** | **95% CI** | | **N** | **Mean** | | **± SD** | | **Median** | | **95% CI** | | **Mean** | **± SD** | **Median** | | **95% CI** | |
| **Diagnosis: Common illness** (without psychosomatic illnesses) | | | | | | | |  |  | |  | |  | |  |  |  |  |  | |  |  |
| Total costs | 33 | | 10,061 | ± 12,570 | 4,589 | [5,604 ; | 14,518] | 2,468 | 3,001 | | ± 8,759 | | 882 | | [2,655 ; | 3,347] | 7,061 | ± 11,580 | 3,082 | | [2,955 ; | 11,167] |
| Ø costs / year observed | 33 | | 4,088 | ± 4,772 | 1,557 | [2,396 ; | 5,780] | 2,468 | 1,163 | | ± 5,607 | | 364 | | [941 ; | 1,384] | 2,925 | ± 4,408 | 977 | | [1,362 ; | 4,488] |
| Ø costs / years insured | 33 | | 3,944 | ± 4,714 | 1,526 | [2,272 ; | 5,615] | 2,468 | 1,107 | | ± 4,312 | | 348 | | [937 ; | 1,278] | 2,836 | ± 4,224 | 946 | | [1,338 ; | 4,334] |
| **Diagnosis: Psychosomatic illness** | | | | | | | |  |  | |  | |  | |  |  |  |  |  | |  |  |
| Total costs | 16 | | 56,584 | ± 106,258 | 9,165 | [-37 ; | 113,205] | 1,199 | 4,179 | | ± 8,936 | | 1554 | | [3,673 ; | 4,685] | 52,407 | ± 105,179 | 5,845 | | [-3,639 ; | 108,453] |
| Ø costs / year observed | 16 | | 12,048 | ± 18,587 | 3,547 | [2,144 ; | 21,953] | 1,199 | 1,616 | | ± 4,827 | | 506 | | [1,342 ; | 1,889] | 10,434 | ± 18,839 | 1,468 | | [395 ; | 20,472] |
| Ø costs / years insured | 16 | | 11,681 | ± 18,052 | 3,619 | [2,062 ; | 21,300] | 1,199 | 1,392 | | ± 3,698 | | 452 | | [1,182 ; | 1,602] | 10,290 | ± 18,195 | 1,920 | | [594 ; | 19,986] |
| **Diagnosis: none** | | | | | | | |  |  | |  | |  | |  |  |  |  |  | |  |  |
| Total costs | 881 | | 28,031 | ± 59,710 | 9,675 | [24,083 ; | 31,980] | 65,331 | 3,689 | | ± 16,991 | | 1132 | | [3,559 ; | 3,819] | 24,358 | ± 59,515 | 6,044 | | [20,422 ; | 28,293] |
| Ø costs / year observed | 881 | | 13,882 | ± 43,193 | 3,276 | [11,026 ; | 16,738] | 65,331 | 1,334 | | ± 7,726 | | 391 | | [1,275 ; | 1,393] | 12,529 | ± 42,900 | 2,090 | | [9,692 ; | 15,365] |
| Ø costs / years insured | 881 | | 20,754 | ± 79,240 | 3,387 | [15,514 ; | 25,994] | 65,331 | 1,651 | | ± 13,872 | | 405 | | [1,544 ; | 1,757] | 19,054 | ± 78,461 | 2,165 | | [13,866 ; | 24,242] |
| **Diagnosis: Rare disease** | | | | | | | |  | |  | |  | |  |  |  |  |  | |  |  |  |
| Total costs | | 313 | 24,367 | ± 53,470 | 7,150 | [18,420 ; | 30,313] | 23,080 | | 3,226 | | ± 15,518 | | 984 | [3,026 ; | 3,426] | 21,155 | ± 53,021 | | 4,276 | [15,258 ; | 27,052] |
| Ø costs / year observed | | 313 | 12,092 | ± 29,464 | 3,036 | [8,815 ; | 15,368] | 23,080 | | 1,248 | | ± 7,855 | | 366 | [1,146 ; | 1,349] | 10,793 | ± 29,233 | | 1,931 | [7,541 ; | 14,044] |
| Ø costs / years insured | | 313 | 18,300 | ± 65,628 | 3,002 | [11,001 ; | 25,599] | 23,080 | | 1,676 | | ± 17,597 | | 368 | [1,449 ; | 1,903] | 16,547 | ± 64,828 | | 1,960 | [9,338 ; | 23,757] |

*Notes: Here, the overall (total) costs are stratified by type of diagnosis within the TNAMSE project.. The total values refer to the sum of all specific costs incurred during the observation period. Ø costs /year observed represent the respective average costs per year of observation. Ø costs /year insured takes into account the actual number of days an individual was insured in the respective observation year. Costs in Euro.*

Appendix - B

Both outpatient services (EBM codes) and inpatient services (OPS codes) were used for **the number of “high-priced” diagnostic services (imaging procedures, genetic examinations, laboratory diagnostics, etc.)**.

The following GOP’s were considered for the analyses reported here:

- Coagulation tests: 32215, 32217, 32219, 32220, 32221, 32222, 32223, 32224, 32225, 32226, 32228, 32229
- Clinical chemistry tests: 32251, 32252, 32291, 32300, 32308, 32311, 32314, 32371, 32374, 32379, 32386, 32388, 32393, 32398, 32402, 32421
- Immunological tests: 32472, 32476, 32504, 32509, 32532, 32533
- Infection immunology tests: 32599, 32640, 32660, 32661, 32670
- Bacteriological tests: 32747, 32764, 32765
- Virological tests: 32783, 32792
- Molecular biological tests: 32835
- Molecular genetic tests: 32860, 32861, 32863, 32864
- General immunogenetic tests: 32931, 32932, 32935, 32940, 32941, 32942, 32943, 32945, 32946, 32947, 32948, 32949
- Histological or cytological examination: 19313, 19314, 19320, 19321, 19330, 19332
- Genetics in connection with pregnancy: 01791, 01792, 01793, 01836, 01837, 01838, 01839, 08571, 08572, 08573, 08574

The following GOP and OPS codes were used for imaging procedures, biopsies and genetic procedures:

| **Procedure** | **GOP starting with:** | **OPS starting with:** |
| --- | --- | --- |
| **CT** | 343 | 3-20 … 3-26 |
| **MRI** | 344 | 3-80 … 3-84 |
| **PET** | 347 | 3-74 |
|  |  | 3-75 |
| **SPECT** | 17362 | 3-72 |
|  |  | 3-73 |
| **Scintigraphy** | 17310 | 3-70 |
|  | 17311 |  |
|  | 17320 |  |
| **Biopsy** | 01755 | 1-40 … 1-49 |
|  | 04517 | 1-50 … 1-58 |
